# Common G-Quadruplex Binding Agents Found to Interact With i-Motif-Forming DNA: Unexpected Multi-Target-Directed Compounds

**DOI:** 10.3389/fchem.2018.00281

**Published:** 2018-07-24

**Authors:** Alessia Pagano, Nunzia Iaccarino, Mahmoud A. S. Abdelhamid, Diego Brancaccio, Emanuele U. Garzarella, Anna Di Porzio, Ettore Novellino, Zoë A. E. Waller, Bruno Pagano, Jussara Amato, Antonio Randazzo

**Affiliations:** ^1^Department of Pharmacy, University of Naples Federico II, Naples, Italy; ^2^School of Pharmacy, University of East Anglia, Norwich Research Park, Norwich, United Kingdom; ^3^Centre for Molecular and Structural Biochemistry, University of East Anglia, Norwich Research Park, Norwich, United Kingdom

**Keywords:** G-quadruplex, i-motif, Berberine, BRACO-19, Mitoxantrone, Phen-DC3, Pyridostatin, RHPS4

## Abstract

G-quadruplex (G4) and i-motif (iM) are four-stranded non-canonical nucleic acid structural arrangements. Recent evidences suggest that these DNA structures exist in living cells and could be involved in several cancer-related processes, thus representing an attractive target for anticancer drug discovery. Efforts toward the development of G4 targeting compounds have led to a number of effective bioactive ligands. Herein, employing several biophysical methodologies, we studied the ability of some well-known G4 ligands to interact with iM-forming DNA. The data showed that the investigated compounds are actually able to interact with both DNA *in vitro*, thus acting *de facto* as multi-target-directed agents. Interestingly, while all the compounds stabilize the G4, some of them significantly reduce the stability of the iM. The present study highlights the importance, when studying G4-targeting compounds, of evaluating also their behavior toward the i-motif counterpart.

## Introduction

GC-rich nucleic acids are able to form a variety of non-canonical secondary structures (Zhao et al., 2010; Cerofolini et al., 2014). The best studied of these are G-quadruplexes (G4s), four stranded alternative nucleic acid secondary structures formed from guanine-rich DNA or RNA composed of stacked tetrads of guanines formed by Hoogsteen hydrogen bonding (Burge et al., 2006). Sequences which can form G4s are prevalent within regulatory regions of the genome, particularly within the promoter region of genes (Huppert and Balasubramanian, 2007; Chambers et al., 2015; Bedrat et al., 2016). Good evidence has been provided to support the hypothesis that G4s exist in human cells (Biffi et al., 2013), play a role in human diseases (Haeusler et al., 2014; Maizels, 2015) and can be targeted with ligands to modulate biological functions (Siddiqui-Jain et al., 2002; Lam et al., 2013; Zizza et al., 2016).

More recently, increasing interest is being paid to the i-motif (iM) structure, another four stranded structure which can form in sequences rich in cytosine, composed of two intercalated hairpins, stabilized by hemi-protonated cytosine-cytosine^+^ (C·C^+^) base pairs (Gehring et al., 1993). Putative iM forming sequences also occur throughout the genome (Wright et al., 2016b; Fleming et al., 2017), typically opposing regions which can form G4s, though the sequence requirements for stable formation are somewhat different. Studies on the iM were previously limited based on the assumption that because they are stabilized in slightly acidic conditions they were not physiologically relevant, despite a solid foundation of data indicating that these structures are detectable at neutral pH *in vitro* (Mergny et al., 1995). Over the years, this assumption has been challenged with examples of iMs which can form at neutral pH, at low temperature (Zhou et al., 2010), under conditions of negative superhelicity (Sun and Hurley, 2009), and molecular crowding (Rajendran et al., 2010) conditions. Further examples of sequences which are naturally stable at neutral pH have been found in the genome, initially by investigating sequences which oppose G4s (Brazier et al., 2012), but multiple other examples have followed (Wright et al., 2016b; Fleming et al., 2017; Mir et al., 2017). Just recently further evidence for i-motif formation *in vivo* has been provided by in cell NMR experiments (Dzatko et al., 2018) and the discovery of an antibody that binds i-motif specifically in the nuclei of human cells (Zeraati et al., 2018). The C-rich regions of genomes are of particular interest because cytosine forms part of the basis for epigenetic regulation, and epigenetic modification of cytosine has been found to alter the stability of iM (Bhavsar-Jog et al., 2014; Xu et al., 2015; Wright et al., 2017). Moreover, ligands which bind and stabilize iM have been shown to modulate biological functions (Amato et al., 2014b). For example, stabilization of the iM forming sequence in the human telomere was found to inhibit telomerase activity and interfere with telomere biology (Li et al., 2006; Chen et al., 2012); stabilization of iM forming sequence in the promoter region of BCL2 was found to cause an increase in gene expression (Kang et al., 2014; Kendrick et al., 2014) and an iM interacting compound was found to downregulate PDGFR-β promoter activity (Brown et al., 2017).

**Figure 1:**
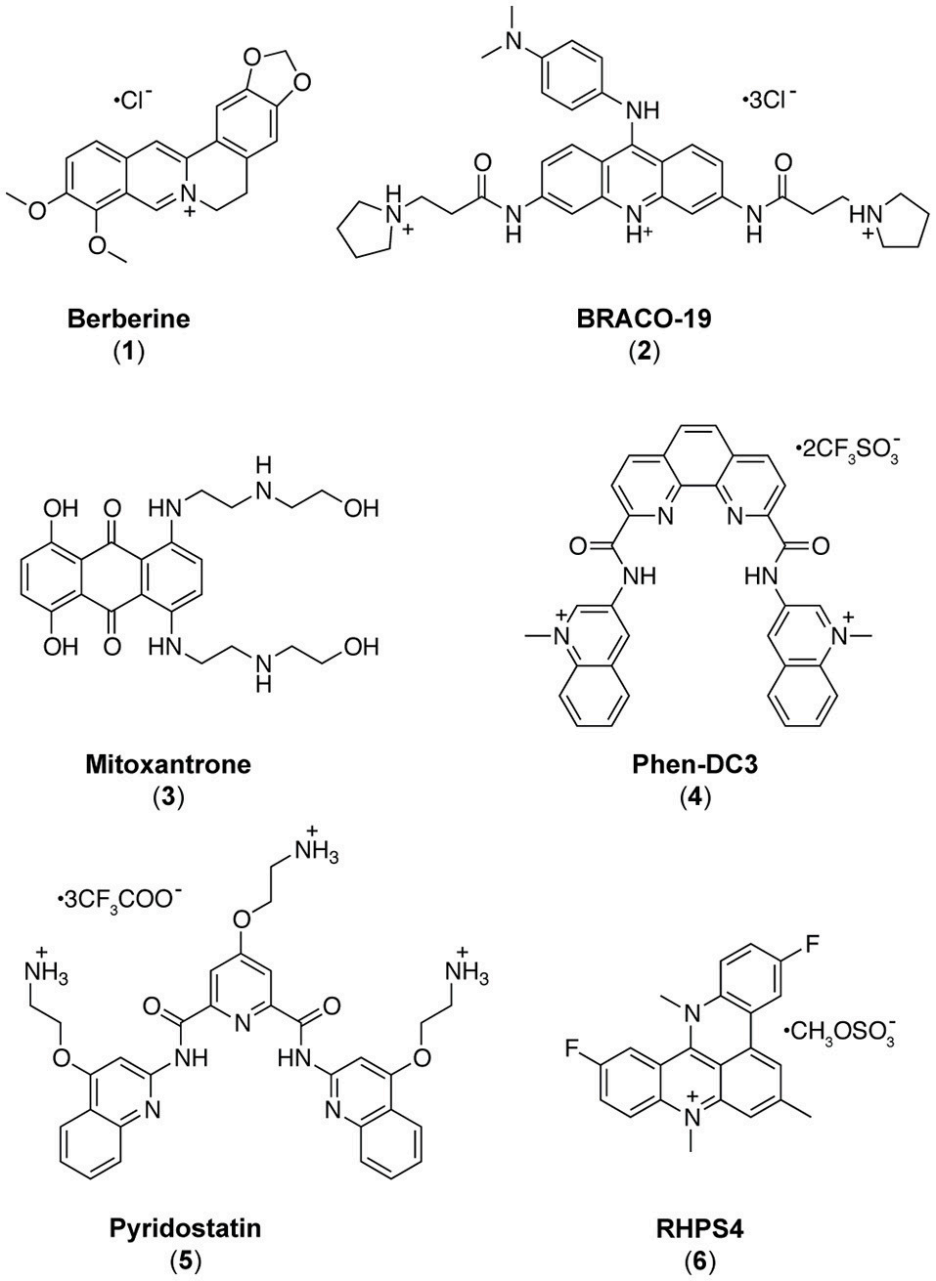
Chemical structures of the investigated ligands.

**Figure 2:**
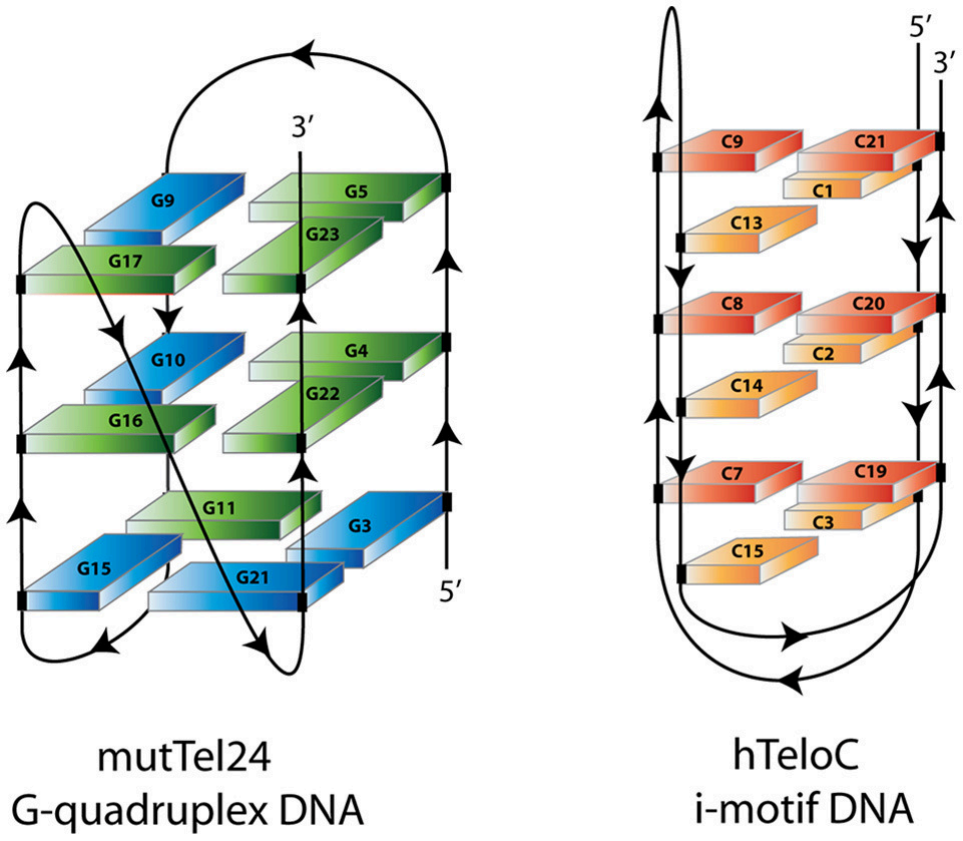
Schematic representation of mutTel24 G-quadruplex and hTeloC i-motif structures. Blue and green solids represent guanines in *syn* and *anti* glycosidic conformation. Orange and red solids represent hemiprotonated C-C^+^ base pairs.

As it appears that formation of iM and/or G4 structures could incite different biological outcomes, it is important to understand the potential structures a compound is able to interact with. In contrast to the hundreds of G4 binding ligands (Pagano et al., 2007, 2010, 2015; Di Leva et al., 2013; Li et al., 2013; Amato et al., 2014a, 2018), there are comparatively very few iM binding compounds reported in the literature (Day et al., 2014). Some ligands which were described to bind G4 have also been found to bind iM (Fedoroff et al., 2000; Wright et al., 2016a; Xu et al., 2016), so we decided to assess and compare the capability to interact with iM-forming DNA of several known bioactive G4 binding agents: Berberine (**1**) (Franceschin et al., 2006), BRACO-19 (**2**) (Gowan et al., 2002), Mitoxantrone (**3**) (Huang et al., 2007), Phen-DC3 (**4**) (De Cian et al., 2007a), Pyridostatin (**5**) (Rodriguez et al., 2008), and RHPS4 (**6**) (Izbicka et al., 1999) (Figure 1). The interaction of these compounds with G4- and iM-forming sequences were investigated *in vitro* in different experimental conditions employing several biophysical methodologies (Pagano et al., 2012) (Figure 2). The data unequivocally demonstrate that, even if in different ways, actually these molecules interact with both DNA, thus acting *de facto* as multi-target-directed compounds.

## Materials and methods

### Oligonucleotide synthesis and sample preparation

An ABI 394 DNA/RNA synthesizer (Applied Biosystem) was employed to prepare DNA sequences at 5-μmol scale by using standard ß-cyanoethylphosphoramidite solid phase chemistry. The subsequent detachment of DNA from support and its deprotection were carried out by means of an aqueous solution of concentrated ammonia at 55°C for 12 h. The filtrates and the washings were combined and concentrated under reduced pressure, solubilized in water, and then purified by high-performance liquid chromatography (HPLC) equipped with a Nucleogel SAX column (Macherey-Nagel, 1000-8/46). Two buffers were employed for the purification: buffer A, consisting of a 20 mM KH_2_PO_4_/K_2_HPO_4_ aqueous solution (pH 7.0) and containing 20% (v/v) CH_3_CN, and buffer B, consisting of 1 M KCl, 20 mM KH_2_PO_4_/K_2_HPO_4_ aqueous solution (pH 7.0), containing 20% (v/v) CH_3_CN, combined in a 30 min linear gradient going from 0 to 100% B with a flow rate of 1 mL/min. The purified fractions of the oligomers were then desalted by using C-18 cartridges (Sep-pak). The purity of the isolated oligomer was evaluated by NMR and it turned out to be higher than 98%. In particular, the following oligonucleotides were employed for the experiments: d[CCCT(AACCCT)_3_] (hTeloC) and d[(TT(GGGTTA)_3_GGGA)] (mutTel24). The oligonucleotide concentrations were established by measuring the UV absorption at 90°C taking into account the molar extinction coefficient values ε (λ = 260 nm) determined by the nearest neighbor model (Cantor et al., 1970). hTeloC and mutTel24 were dissolved in 10 mM sodium phosphate buffer and 10 mM potassium phosphate buffer, respectively, at different pH values before establishing the experimental conditions to be used (pH 4.3 and pH 5.7). DNA samples were heated at 90°C for 5 min, and then gradually cooled to room temperature overnight.

### Circular dichroism spectroscopy

Circular dichroism (CD) experiments were recorded on a Jasco J-815 spectropolarimeter equipped with a PTC-423S/15 Peltier temperature controller. Each spectrum was recorded in the 220–360 nm wavelength range, averaged over three scans and subtracted of the buffer baseline. The scan rate was set to 100 nm/min, with a 1 s response time, and 1 nm bandwidth. Spectra were analyzed using Origin 7.0 software. CD experiments (spectra and melting) were performed using 10–15 μM oligonucleotide concentration, in the absence and presence of 5 molar equivalents of ligands (10 mM in DMSO). CD melting were performed at 1°C/min heating rate in the 5–90 and 20–100°C temperature range for hTeloC and mutTel24, respectively. Changes of CD signal were followed at the wavelengths of the maximum CD intensity, 288 and 290 nm for hTeloC and mutTel24, respectively. The melting temperatures (*T*_1/2_) were mathematically calculated by using the curve fitting function in Origin 7.0 software. Δ*T*_1/2_ values represent the difference between the melting temperature of the DNA with and without ligands.

### UV-melting

A JASCO V-730 UV-visible spectrophotometer equipped with a Peltier temperature controller was employed to perform the UV thermal denaturation experiments. The oligonucleotide concentrations were 10 μM for both hTeloC and mutTel24 DNA in the appropriate buffer, as indicated above. Experiments were performed by following changes of UV signal at 295 nm, at a heating rate of 1°C/min, in the temperature ranges of 5–100 and 20–100°C for hTeloC and mutTel24, respectively. The melting temperatures (*T*_1/2_) were mathematically calculated by using the curve fitting function in Origin 7.0 software. Δ*T*_1/2_ values represent the difference between the melting temperature of the DNA with and without ligands.

### Nuclear magnetic resonance experiments

A 700 MHz Varian Unity INOVA spectrometer was employed to perform the NMR experiments. One-dimensional proton spectra were recorded at 7°C using pulsed-field gradient DPFGSE for water suppression. All DNA samples were prepared at 0.2 mM strand concentration in 0.22 mL (H_2_O/D_2_O 9:1) buffer solution. DNA/ligand mixtures were obtained by adding aliquots of a stock solution of the six ligands in DMSO-d6 directly to the DNA solution inside the NMR tube (Randazzo et al., 2002; Amato et al., 2017). NMR data were processed using the iNMR software (www.inmr.net).

### FRET and FRET-melting

A FP-8300 spectrofluorometer (Jasco) equipped with a Peltier temperature controller accessory (Jasco PCT-818) was employed to carry out FRET experiments. The dual-labeled oligonucleotides corresponding to the G4 forming sequence 5′-FAM-d(GGG[TTAGGG]_3_)-TAMRA-3′ (G4-F21T) (Amato et al., 2016; Salvati et al., 2017) and the iM forming sequence 5′-FAM-d(TAACCC)_4_-TAMRA-3′ (iM-F24T) were used. Such sequences are characterized by the presence of the donor fluorophore FAM (6-carboxyfluorescein) and the acceptor fluorophore TAMRA (6-carboxytetramethylrhodamine) that are covalently bound at 5′- and 3′-ends, respectively. Labeled oligonucleotides were purchased from Biomers (Germany). G4-F21T and iM-F24T were prepared at 1 μM concentration in 10 mM potassium phosphate buffer and 10 mM sodium phosphate buffer, respectively. Samples were annealed in a hot water bath at 90°C for 2 min, and then cooled to room temperature overnight. FRET measurements were performed both in the absence and presence of 5 molar equivalents of compounds **1**-**6**. The final concentration of G4-F21T and iM-F24T was 0.1 μM. A sealed quartz cuvette with a path length of 1 cm was used. FRET spectra were acquired before (at 5 and 20°C for iM and G4, respectively) and after (at 90°C) melting assay. The dual-labeled oligonucleotides were excited at 492 nm, and emission spectra were recorded between 500 and 650 nm using 100 nm/s scan speed. Excitation and emission slit widths were both set at 5 nm. FRET-melting experiments were performed by setting the excitation wavelength at 492 nm and the detection wavelength at 522 nm. The emission intensity of FAM was then normalized between 0 and 1. Data analysis was carried out using Origin 7.0 software.

### Fluorescent intercalator displacement (FID) assay

For the FID experiments, oligonucleotides d[(TAACCC)_4_] (hTeloC_FID_) and d[(TTTGGG(TTAGGG)_3_A)] (mutTel24_FID_) were purchased from Eurogentec and then purified via HPLC. Solid DNA samples were initially dissolved as a 1 mM stock solution in MilliQ water. 10 mM stock solutions of the candidate ligands were prepared in DMSO. Further dilutions were carried out in buffer: 10 mM NaH_2_PO_4_ for hTeloC_FID_ and 10 mM KH_2_PO_4_ for mutTel24_FID_. DNA samples were thermally annealed at 90 μM in the respective buffers in an Applied Biosystems Veriti 96 well thermal cycler by holding at 95°C for 5 min and cooling at a rate of 1°C/min to 20°C. FID experiments were performed on a BMG CLARIOstar plate reader using Corning 96-Well Solid Black Flat Bottom plates. A 10 mM stock solution of thiazole orange (TO) was prepared in DMSO and diluted to 2 μM in the appropriate buffer for either hTeloC_FID_ or mutTel24_FID_. Ninety microliters of the 2 μM TO solution were added to each well and fluorescence emission at 450 nm measured with excitation at 430 nm; this was normalized to 0% representing background fluorescence. One microliter of DNA was then added, shaken using double orbital shaking at 700 rpm in the plate reader for 15 s, and allowed to equilibrate for 15 min. After equilibration, fluorescence emission was measured as before, and normalized to 100% representing maximal fluorescence enhancement from the TO probe binding to the DNA secondary structure. 0.9 μL aliquots of ligand were titrated into each well (in triplicate) and measured as before. Fluorescence measurements after ligand addition were normalized between the 0 and 100% levels determined per the respective well. Percentage TO displacement was calculated as the difference between the normalized 100% fluorescence level and the normalized fluorescence measured after each ligand addition. The concentration for each ligand at which 50% of the TO was displaced (DC_50_) was calculated by using Origin data analysis software to plot percentage TO displacement against ligand concentration. These data were fitted with dose-response curves and the equations of the curves were solved for y = 50 to give the DC_50_ values.

## Results

Most of the investigations reported in the literature dealing with the determination of iM structures in solution have been accomplished in sodium buffer and under acidic conditions, generally at pH values down to 4.3 (Gehring et al., 1993; Gallego et al., 1997; Malliavin et al., 2003). This is because the cytidine pK_a_ is about 4.2, and the use of low pH values guarantees to obtain stable hemi-protonated C·C^+^ pairs. However, these conditions are far away from physiological. This may also have consequences on the study of the interaction between these target molecules and potential ligands, which may be differently protonated with respect to their state under physiological pH. Therefore, in order to find experimental conditions as close to physiological as possible, the behavior of the telomeric iM-forming sequence (hTeloC) in 10 mM sodium buffer at different pH values was investigated by 1D ^1^H-NMR and CD spectroscopies (Figures S1, S2, Supplementary Material). In particular, the pH range 4.3–7.0 was explored. NMR and CD spectra clearly indicated that the hTeloC sequence turned out to be folded into an iM structure only at pH values lower than 5.7, while above this value the iM structure is in equilibrium with the unfolded species. Therefore, we decided to perform our studies at the two boundaries pH values, namely 4.3 and 5.7. Since the main aim of this investigation was to evaluate the interaction of some known G4 ligands with an iM-forming DNA, experiments on the human telomeric G4 (mutTel24) were performed in parallel for comparison.

### Circular dichroism studies

**Figure 3:**
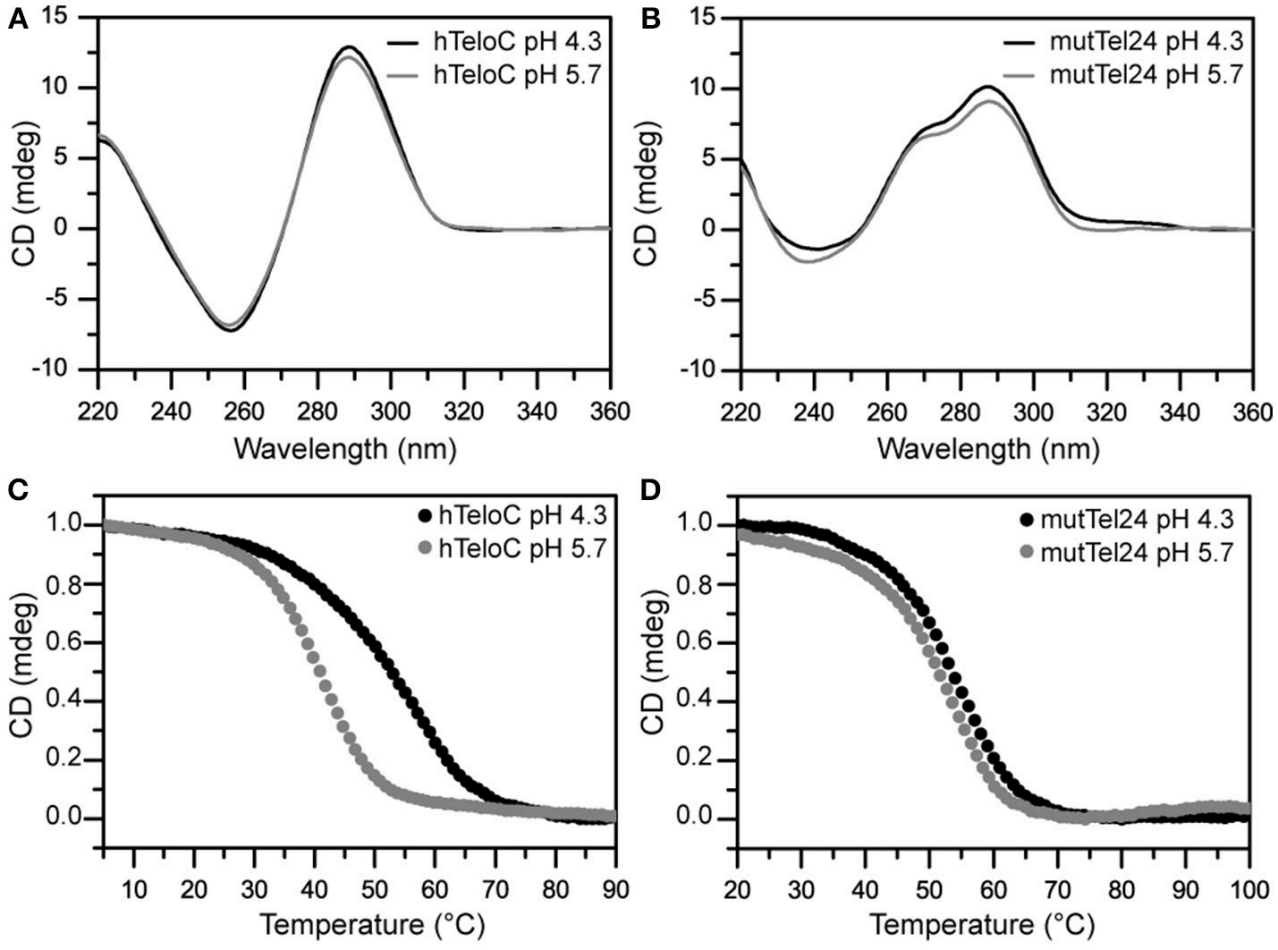
**(A,B)** CD spectra of hTeloC and mutTel24 at pH 4.3 (black) and 5.7 (gray). **(C,D)** CD melting of hTeloC and mutTel24 at pH 4.3 (black) and 5.7 (gray).

The structures adopted by hTeloC and mutTel24 were first investigated by circular dichroism (CD) spectroscopy in the absence of ligands. At both pH 4.3 and 5.7, hTeloC showed almost superimposable CD spectra having a positive band at 288 nm and a negative one at around 260 nm (Figure 3A). These bands are characteristic of an iM folding topology (Guo et al., 2008). mutTel24 also displayed almost superimposable CD spectra at both pH values. These spectra were characterized by two positive bands at around 290 and 270 nm, and a negative one at around 240 nm (Figure 3B). These bands are perfectly superimposable to those observed for the same molecule at pH 7.0, thus indicating the presence of the expected hybrid [3+1] G4 structure (hybrid-1) as the major conformation under acidic conditions (Karsisiotis et al., 2011; Gray et al., 2014).

**Figure 4:**
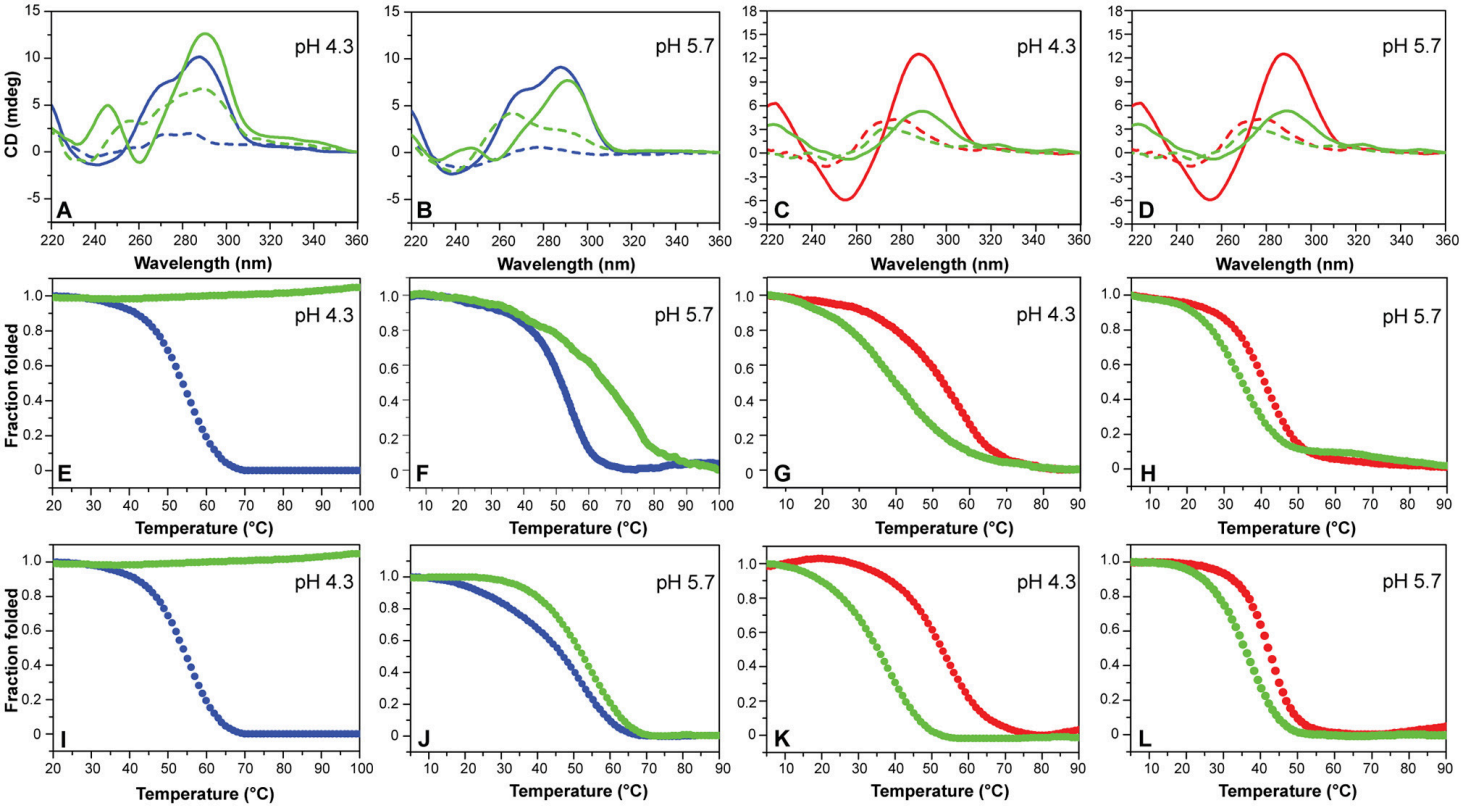
**(A,B)** CD spectra of mutTel24 in the absence (blue) and presence (green) of 5 equivalents of Phen-DC3 (**4**) at 20°C (solid lines) and 100°C (dashed lines). **(C,D)** CD spectra of hTeloC in the absence (red) and presence (green) of 5 equivalents of **4** at 5°C (solid lines) and 100°C (dashed lines). **(E,F)** CD- and **(I,J)** UV-melting of mutTel24 in the absence (blue) and presence (green) of 5 equivalents of **4**. **(G,H)** CD- and **(K,L)** UV-melting of hTeloC in the absence (red) and presence (green) of 5 equivalents of **4**. The relative pH values are reported in each panel.

CD experiments were also performed to examine the potential of compounds **1**-**6** to alter the native folding topology of the two investigated DNA structures both at pH 4.3 and 5.7. DNA/ligand mixtures were obtained by adding 5 molar equivalents of compound to the folded G4 and iM structures so as to have an excess with respect to potential binding sites. In the case of mutTel24, regardless of the pH, Berberine (**1**), BRACO-19 (**2**), Phen-DC3 (**4**) and RHPS4 (**6**) induced a significant change in the CD spectrum of the G4 structure (Figures 4A,B and Figures S3, S4, Supplementary Material). In particular, the loss of the band at 270 nm followed by an intensity's increase of the band at 290 nm suggested a conformational change of the G4 topology from the hybrid to the antiparallel conformation (Masiero et al., 2010; Randazzo et al., 2013). Conversely, Mitoxantrone (**3**) and Pyridostatin (**5**) did not produce any measurable conformational change of the G4 structure, even if a decrease of the band at 290 nm is observed upon their addition.

As for the iM structure, at pH 4.3, Mitoxantrone (**3**), Phen-DC3 (**4**), and RHPS4 (**6**) induced a significant hypochromic shift of the positive band at 288 nm, with more marked effects observed for **3** and **4**. Conversely, Berberine (**1**), BRACO-19 (**2**), and Pyridostatin (**5**) caused a hyperchromic shift of the band at 288 nm at this pH (Figure 4C and Figure S5, Supplementary Material). On the other hand, at pH 5.7 all the compounds have been shown to induce a hypochromic effect of the band at 288 nm that turned out to be particularly marked in the case of **2**, **3**, and **4** (Figure 4D and Figure S6, Supplementary Material). These data suggest that some interaction takes place and that, in some cases, the molecules seem to induce the unfolding of the structure.

### CD-melting analysis

CD-melting experiments were employed to evaluate the thermal stability of iM and G4 structures adopted by hTeloC and mutTel24 sequences, respectively, under the two experimental conditions used (pH 4.3 and 5.7). The melting temperatures (*T*_1/2_) of the iM structure were found to be 52.8 and 40.6°C at pH 4.3 and 5.7, respectively (Figure 3C). The lower thermal stability of the iM structure observed at pH 5.7 can be ascribed to the lower extent of protonation of the cytosines. On the other hand, only a very small variation in *T*_1/2_ values was observed for the G4 structure between pH 4.3 and 5.7 (53.5 and 51.1°C, respectively, Figure 3D).

Then, the effect of the investigated compounds on the stability of the DNA secondary structures was evaluated by measuring the ligand-induced change in the melting temperature (Δ*T*_1/2_) of G4 and iM at pH 4.3 and 5.7. CD-melting curves of DNA in the absence and presence of each compound were obtained by following the variations of the intensity of CD signal at the wavelengths of 290 and 288 nm for mutTel24 and hTeloC, respectively (Figure 4 and Figures S7–S10, Supplementary Material). Very intriguingly, results of these experiments (Table 1) clearly indicated that **1**-**6** exert a different effect on iM compared to G4. As expected, all the tested compounds were able to thermally stabilize the G4 structure at both pH values, even if to a different extent (Figures 4E,F, and Figures S7, S8, Supplementary Material). On the contrary, regardless of pH, Berberine (1), Pyridostatin (5), and RHPS4 (6) did not show a remarkable influence on the iM thermal stability, while BRACO-19 (2), Mitoxantrone (3), and Phen-DC3 (4) significantly decreased it (Figures 4G,H and Figures S9, S10, Supplementary Material). These results are in agreement with those obtained from the CD spectra analysis.

**Table 1:** Ligand-induced thermal stabilization of hTeloC and mutTel24 DNA measured by CD and UV melting experiments.

	Δ*T*_1/2_ (°C)^*^ CD melting				Δ*T*_1/2_ (°C)^*^ UV melting			
	**hTeloC**		**mutTel24**		**hTeloC**		**mutTel24**	
	**pH 4.3**	**pH 5.7**	**pH 4.3**	**pH 5.7**	**pH 4.3**	**pH 5.7**	**pH 4.3**	**pH 5.7**
Berberine	−2.5	−0.8	+13.4	+12.4	+1.2	+0.9	+11.7	+15.3
BRACO-19	−13.4	−9.2	+12.4	+8.9	−17.6	−6.5	*ND*	*ND*
Mitoxantrone	−4.8	−9.9	+7.7	+4.5	−16.6	−1.0	+4.1	+0.6
Phen-DC3	−13.4	−6.8	*ND*	+14.4	−17.1	−6.3	*ND*	+5.3
Pyridostatin	−2.8	+0.8	+12.9	+8.8	+1.1	+0.2	+9.3	+5.5
RHPS4	−1.0	−0.3	+22.0	+20.6	+0.7	+0.1	*ND*	*ND*

* *ΔT1/2 = T1/2(DNA+ligand) -T1/2(DNA). All experiments were performed in duplicate, and ΔT1/2 values are reported as the mean. Errors were ±0.5°C. ND, not determined*.

### UV-melting analysis

The effect of compounds **1**-**6** on the stability of the G4 and iM structures was also investigated by UV-melting experiments. As for CD-melting studies, the ligand-induced changes in the melting temperature (Δ*T*_1/2_) of the two DNA structures were obtained by recording UV-melting experiments in the absence and presence of each compound at both pH 4.3 and 5.7. UV-melting curves were acquired by following the change in UV signal intensity at 295 nm for both mutTel24 and hTeloC (Figures 4I–L, and Figures S11–S14, Supplementary Material). Results of these experiments (Table 1) are consistent with CD-melting ones, and denote, once again, a different behavior for the investigated compounds toward the iM and G4 DNA structures.

### Nuclear magnetic resonance studies

NMR spectroscopy was employed in order to obtain structural information about the DNA interaction of the six compounds. Also in this case, 1D ^1^H-NMR spectra were recorded at pH 4.3 and 5.7. Under the experimental conditions used, the mutTel24 sequence forms a single G4 conformation characterized by 12 well-resolved imino proton peaks, corresponding to the 12 guanines involved in the three G-tetrad planes (Luu et al., 2006). On the other hand, hTeloC folds in an i-motif structure characterized by 3 well-resolved imino proton peaks that correspond to the 6 intercalated C·C^+^ pairs (Phan et al., 2000).

**Figure 5:**
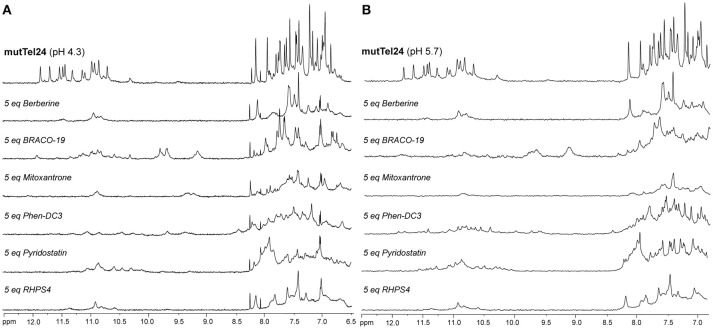
^1^H-NMR spectra of mutTel24 in 10 mM KH_2_PO_4_ at **(A)** pH 4.3 and **(B)** pH 5.7, before and after the addition of 5 equivalents of each ligand.

**Figure 6:**
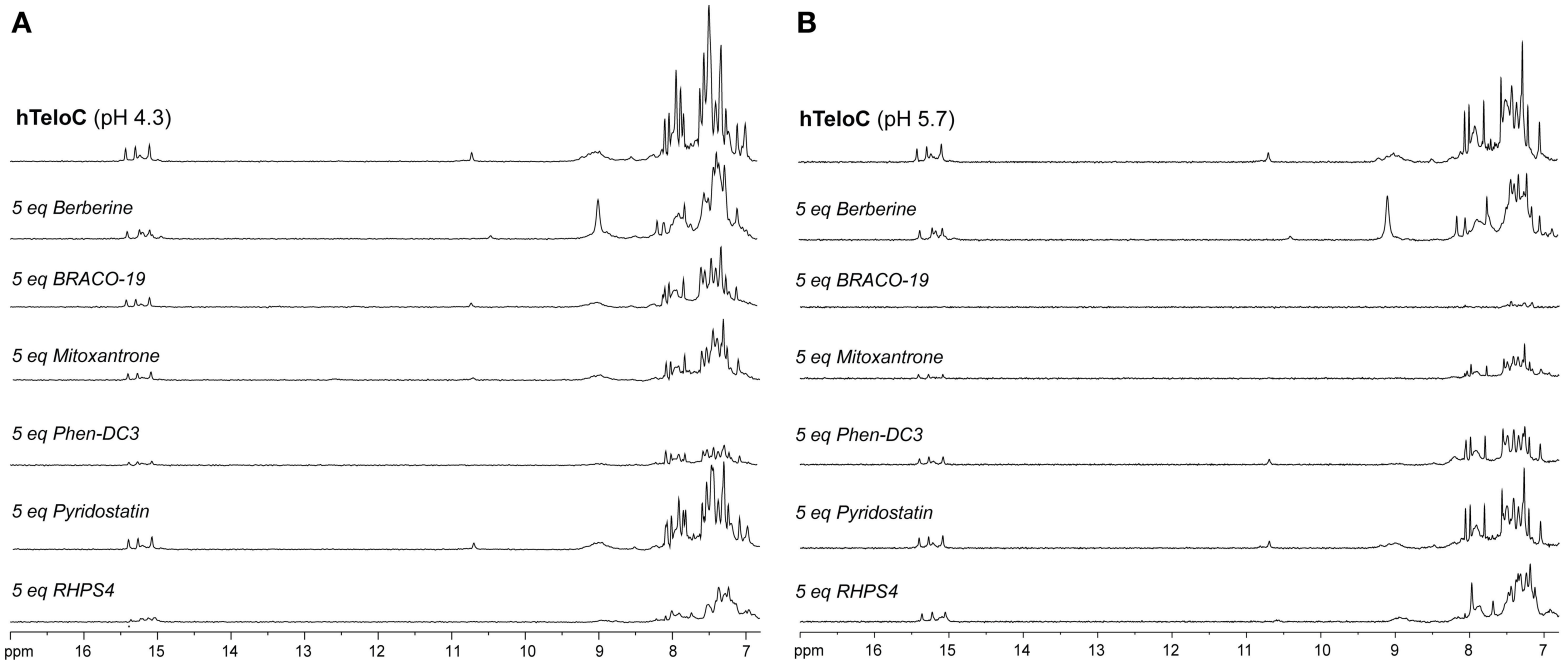
^1^H-NMR spectra of hTeloC in 10 mM NaH_2_PO_4_ at **(A)** pH 4.3 and **(B)** pH 5.7, before and after the addition of 5 equivalents of each ligand.

The imino and aromatic proton regions of mutTel24 and hTeloC in the absence and presence of 5 equivalents of each compound are shown in Figures 5, 6, respectively. Regardless of the pH, both imino and aromatic proton signals of mutTel24 turned out to be significantly affected by the addition of the ligands (Figure 5). On the other hand, addition of compounds to the iM structure led to different results. At pH 4.3, the main changes were observed for Berberine (**1**) and RHPS4 (**6**) in both aromatic and imino regions, while little changes could be observed for BRACO-19 (**2**) and Mitoxantrone (**3**). Instead, Phen-DC3 (**4**) caused a general decrease of signal intensities (Figure 6A). Very little changes could be observed in the spectrum of iM upon addition of Pyridostatin (**5**). At pH 5.7, changes were observed for Berberine (**1**) BRACO-19 (**2**), Mitoxantrone (**3**), and RHPS4 (**6**) (Figure 6B), with BRACO-19 affecting the most the NMR spectrum of the iM structure. Interstingly, as for the experiment at pH 4.3, PhenDC3 (**4**) and Pyridostatin (**5**) caused a general decrease of the signal intensities.

### FRET and FRET-melting studies

Dual labeled (FAM/TAMRA) human telomeric sequences G4-F21T and iM-F24T, which are able to form G4 and iM structures, respectively, were used (see Experimental section). Experiments were performed only at pH 5.7, because the fluorescence of the FAM is not stable at pH 4.3. In order to verify that **1**-**6** did not interfere in the FAM emission spectrum, the fluorescence spectrum of each compound was recorded by exciting at 492 nm and collecting its emission spectrum between 500 and 650 nm. Unfortunately, the emission spectrum of RHPS4 (**6**) was found to overlap with FAM, and, therefore, it was not used in these experiments.

FRET-melting assays were then performed to further investigate the compound-induced effects on the iM and G4 thermal stabilities. In agreement with CD- and UV-melting experiments, all the tested compounds induced a thermal stabilization of G4-F21T (Figure S15, Supplementary Material). Conversely, in the case of iM-F24T, FRET melting data did not agree at all with CD and UV melting results. In fact, all ligands, except Berberine, showed a significant thermal stabilization of the iM structure (Figure S16, Supplementary Material).

**Figure 7:**
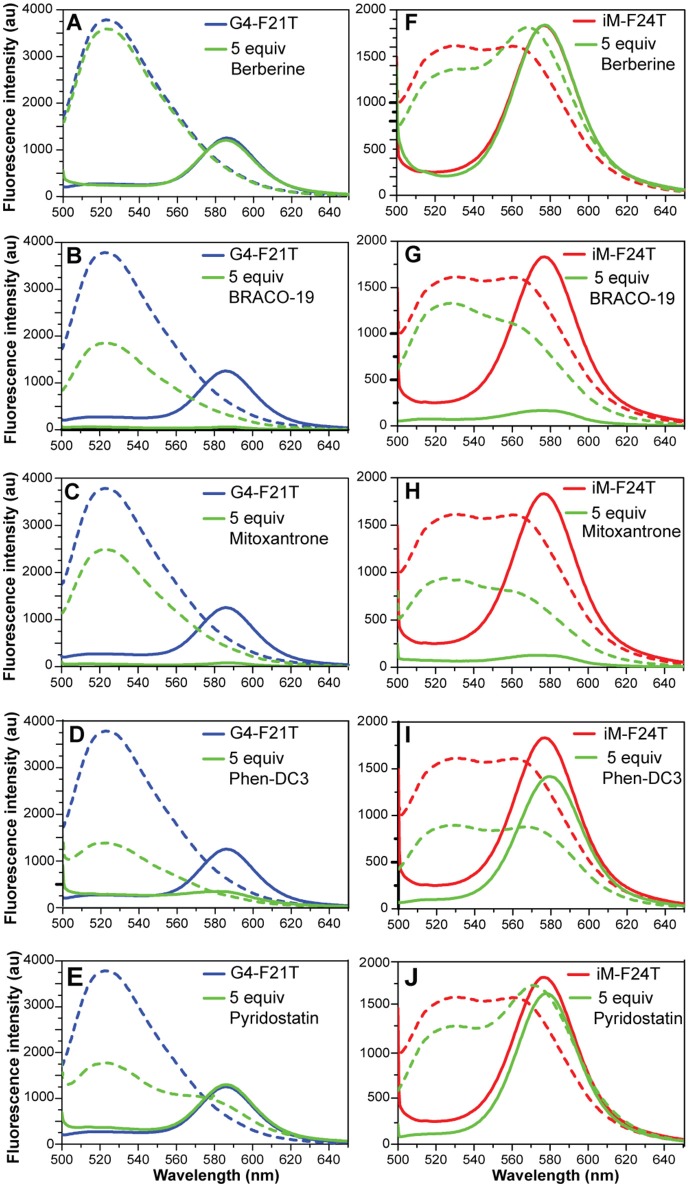
**(A–E)** Fluorescence spectra of G4-F21T alone (in 10 mM KH_2_PO_4_ buffer) at 5°C (blue line) and at 90°C (dashed blue line), and after the addition of 5 equivalents of each ligand at 5°C (green line) and at 90°C (dashed green line). **(F–J)** Fluorescence spectra of iM-F24T alone (in 10 mM NaH_2_PO_4_ buffer) at 5°C (red line) and at 90°C (red dashed line), and after the addition of 5 equivalents of each ligand at 5°C (green line) and at 90°C (dashed green line). All the experiments were performed at pH 5.7.

FRET spectra of FAM/TAMRA-modified oligonucleotides (G4-F21T and iM-F24T) in the absence and presence of each compound (at 1:5 DNA/ligand ratio) were also analyzed (Figure 7). The results show that, in both cases, BRACO-19 (**2**), Mitoxantrone (**3**), Phen-DC3 (**4**), and Pyridostatin (**5**) caused a significant decrease of the band intensity at 580 nm, thus suggesting that actually they could interact with the probes.

### Fluorescent intercalator displacement (FID) assay

FID assays are long established and have been used extensively to determine relative binding affinities for ligands with duplex DNA (Boger et al., 2000). They have recently been adapted and validated for use with both G4 and iM structures (Monchaud et al., 2006; Sheng et al., 2017). The assay relies on a light-up fluorescent probe, in this case thiazole orange (TO), which binds to the structure of interest and can be competitively displaced by candidate ligands thus enabling the determination of their relative binding affinity to the structure under examination. Here, aliquots of ligands **1-5** were titrated in triplicate against both of the G4 and iM structures and the concentrations at which 50% displacement (DC_50_) was achieved were calculated from dose-response curves fitted to this data (Figures S17, S18, Supplementary Material). Unfortunately, as with the FRET, RHPS4 **(6)** was excluded from analysis due to the fluorescence profile of the ligand overlapping with the assay parameters. The results of the FID assay showed that all ligands **(1-5)** bound to both the G4 and the iM DNA. Unsurprisingly, they also all showed a slightly higher affinity for the G4 formed by mutTel24_FID_ over the iM formed by hTeloC_FID_ (Table 2). Nevertheless, all ligands tested were found to displace TO at both pH 4.3 and 5.7, indicative of an interaction with the iM-forming DNA.

**Table 2:** Ligand DC_50_ values for hTeloC_FID_ and mutTel24_FID_ determined using the FID assay^*^.

	**hTeloC**_**FID**_				**mutTel24**_**FID**_			
	**pH 4.3**		**pH 5.7**		**pH 4.3**		**pH 5.7**	
	**DC_50_ (μM)**	**SE (μM)**	**DC_50_ (μM)**	**SE (μM)**	**DC_50_ (μM)**	**SE (μM)**	**DC_50_ (μM)**	**SE (μM)**
Berberine	30.38	1.58E-02	1.46	5.88E-03	3.32	2.63E-02	1.26	7.22E-03
BRACO-19	0.66	1.61E-03	0.87	8.08E-04	0.26	5.14E-03	0.50	1.95E-03
Mitoxantrone	0.70	2.87E-03	1.34	6.99E-03	0.54	3.03E-04	0.95	6.07E-03
Phen-DC3	0.97	1.32E-02	0.95	3.79E-03	0.26	1.58E-03	0.39	1.42E-03
Pyridostatin	9.09	6.18E-03	18.02	5.89E-02	3.15	2.33E-03	9.42	3.90E-02

* *All experiments were performed in triplicate and DC_50_ values are reported as the 50% displacement value calculated from fitted dose response curves. Standard errors are calculated using R-square values from the statistics on the data fit*.

## Discussion

DNA has a well-known propensity to adopt various alternative non-canonical conformations *in vitro*, including G4 and iM. Recent investigations have demonstrated the formation of such structures in regulatory regions of the human genome, including gene promoters and telomeres, also providing evidences for the key role that G4s and iMs can play in several biological pathways (Kang et al., 2014; Salvati et al., 2014; Maizels, 2015).

Human telomeric DNA consists of a 2–20 kb double-stranded region composed by (TTAGGG)/(CCCTAA) repeats, and of a single-stranded 3′-end G-rich sequence. The G-rich strand can adopt G4 conformations, while the opposite C-rich strand can fold into the iM structure. Of the two structures, telomeric G4 is by far the most studied. This disparity is mainly due to acidic pH required for the protonation of cytosine, since the parallel duplexes, the basic component of iM, are stabilized by hemiprotonated C·C^+^ base pairs. In this investigation, the behavior of the human telomeric iM-forming sequence (hTeloC) was studied in the pH range of 4.3–7.0 by 1D ^1^H-NMR and CD spectroscopies. Results clearly indicate that under the experimental conditions used, the iM structure is still well-preserved at pH 5.7, while, just above this pH value, it turns out to be in equilibrium with the random coil (Figures S1, S2, Supplementary Material). Moreover, the lower stability of the iM structure observed at pH 5.7 well reflects the lower extent of cytosine protonation compared to pH 4.3, thus confirming, once again, that iM structures are very sensitive to pH (Kovanda et al., 2015). On the other hand, results of NMR and CD experiments on the G-rich telomeric sequence clearly showed that it retains the hybrid [3+1] G4 structure as major conformation at both pH 4.3 and 5.7, and that its thermal stability is basically not affected by the pH.

Once the working conditions were established, the interaction of the well-known G4 ligands Berberine (**1**), BRACO-19 (**2**), Mitoxantrone (**3**), Phen-DC3 (**4**), Pyridostatin (**5**), and RHPS4 (**6**), with the iM-forming sequence was explored in comparison with the G4, by using a combination of spectroscopic techniques. Experiments were performed at the two boundary pH values. Results of the different experiments undoubtedly showed that all the investigated compounds actually are able to interact, even if in a different way, with both G4- and iM-forming DNA.

Fluorescent intercalator displacement (FID) assay clearly showed that the thiazole orange probe, which binds to the investigated structures, is competitively displaced by the compounds **1**-**5** (compound **6** could not be used). The consequent determination of their relative affinity for the DNA under examination reveals that, regardless of the pH (4.3 or 5.7), the compounds exhibit only a slightly higher affinity for mutTel24 over hTeloC.

Ligand-induced effects on both G4 and iM structures were examined by means of UV, CD, and NMR. As far as mutTel24 is concerned, results of CD experiments show that four (Berberine, BRACO-19, Phen-DC3, and RHPS4) out of the six ligands induce a conformational change from the hybrid [3+1] to an antiparallel structure (Figures S3, S4). All the ligands also indiscriminately affect the NMR spectrum of mutTel24 (Figure 5). Results of CD- and UV-melting experiments agree in indicating that the six compounds are able to stabilize the G4 at both pH values (Figures S7, S8, S11, S12, Supplementary Material). Interestingly, the two ligands not inducing the G4 conformational change (Mitoxantrone and Pyridostatin) seem also to be the less effective in terms of thermal stabilization under the experimental conditions used. Moreover, some of the investigated ligands induced a higher increase of G4 melting temperature (Δ*T*_1/2_) at pH 4.3 rather than 5.7 (Table 1), that may be ascribed in part to the different protonation states of such molecules at the different pHs. This was, for example, the case of BRACO-19 (**2**) and Pyridostatin (**5**). For these molecules the protonation state was theoretically determined in the 3.5–7.5 pH range, by using the p*K*_a_ prediction program (ChemAxon, www.chemaxon.com) based on the calculation of partial charge of atoms in the molecule. The computed distribution of the microspecies for BRACO-19 and Pyridostatin, reported in Figure S19, shows that actually there is a variation between pH 4.3 and 5.7 for these molecules.

Concerning the iM, some differences were observed between pH 4.3 and 5.7. In particular, the addition of **1**-**6** to the iM caused major effects at pH 5.7, in which the iM structure is less stable *per se*.

Interestingly, CD- and UV-melting results clearly indicate that the molecules which were found to mainly decrease the CD and NMR signals (namely BRACO-19, Mitoxantrone, and Phen-DC3) were also those that significantly decreased iM thermal stability at both pH values (Figures S9, S10, S13, S14, Supplementary Material).

Overall, these results show that compounds **1**-**6** are able to interact with the telomeric iM-forming DNA. However, three of them (Berberine, Pyridostatin, and RHPS4) do not have relevant effects on the thermal stability of iM, while the others (BRACO-19, Mitoxantrone, and Phen-DC3) are able to destabilize it. We speculate that these compounds could reasonably make some non-specific interactions with the single-stranded (unfolded) C-rich DNA, resulting in a shift of folded-unfolded equilibrium toward the unfolded form, especially during the melting experiment, which in turn results in a decrease of *T*_1/2_.

The FRET methodology, used here to further characterize the interaction of G4 and iM DNA with the investigated ligands, deserves a separate discussion. With respect of other spectroscopic techniques (such as UV, CD, and NMR), FRET has a higher sensitivity and it can explore a large range of ligand concentrations (Monchaud et al., 2006; Sheng et al., 2017). Additionally, it turns out to be the main methodology when the UV absorbance of a ligand overlaps with that of the DNA (Guédin et al., 2010). However, some artifacts may occur when compounds are inherently fluorescent and/or interact with the fluorescent probes rather than the DNA itself (De Cian et al., 2007b), and this is what probably happened in this case. Indeed, FRET melting results did not agree at all with both CD and UV melting data, especially in the case of iM-F24T (Figure S16, Supplementary Material). To understand the reasons for this different behavior, FRET spectra of labeled DNA (G4-F21T and iM-F24T) in the absence and presence of compounds were analyzed. Typically, when DNA is folded, the two dyes are in close proximity so FAM fluorescence peak at 522 nm (upon excitation at 492 nm) is quenched and its energy is transferred to TAMRA, which then emits light at 580 nm. On the other hand, FAM's fluorescence is no longer quenched when sufficient spatial separation of the two dyes occurs (for example upon unfolding of the DNA structure), therefore its fluorescence signal at 522 nm is observable. In principle, compounds that are able to interact with the fluorophores may affect the emission properties of the probes and decrease the intensity of the bands at 580 nm (if the DNA is structured) or at 522 nm (if the DNA is unstructured). Interestingly, four compounds (BRACO-19, Mitoxantrone, Phen-DC3, and Pyridostatin) caused a significant decrease of the band intensity at 580 nm (for both G4-F21T and iM-F24T), clearly suggesting that they actually interact with the fluorophores. Therefore, the different stabilizing effects observed for the iM structure across the different spectroscopic techniques could be ascribed to ligand interaction with the FRET probes. This hypothesis is further corroborated by the fact that Berberine (**1**), which did not cause any observable change in the FRET spectrum, showed no variation in the DNA's thermal stability by FRET, in agreement with CD and UV experiments. For the same reasons, this suggests that the thermal stabilizations of G4-F21T measured by FRET are not accurate, being also potentially affected by the ligands' interaction with the probes. Therefore, a clear message came out from a careful examination of FRET data, meaning that false-positive responses can be obtained due to ligands ability to bind end-labeling DNA probes. This could occur particularly when the investigated compounds have an extended aromatic core for which π-π stacking interactions with the large aromatic surface of the probes could be favored. This is especially the case of G4-interacting molecules. Overall, this study emphasizes the need of using a combination of techniques when examining DNA targeting ligands, in order to avoid an inaccurate evaluation of their binding/stabilizing properties.

## Conclusions

Herein, a combination of spectroscopic techniques was employed to determine whether well-known bioactive G4 ligands, namely Berberine (**1**), BRACO-19 (**2**), Mitoxantrone (**3**), Phen-DC3 (**4**), Pyridostatin (**5**), and RHPS4 (**6**) are able to interact with an iM-forming DNA. Two human telomeric DNA sequences able to form iM and G4 structures were studied and the experiments performed at two different pH values. The experimental results showed that all the investigated G4 ligands were also able to interact with the telomeric iM-forming DNA. Very interestingly, BRACO-19, Mitoxantrone, and Phen-DC3 have been shown to destabilize the iM structure.

The majority of iM forming sequences are generally less stable than G4s under physiological conditions. The delicate equilibrium between the folded and unfolded DNA forms is highly sensitive to the environmental conditions (such as pH and ionic strength), and ligands may affect this fine equilibrium, shifting it toward different/less stable forms.

The here reported results are even more interesting if viewed in the context of regulation of gene expression. Indeed, recent investigations have suggested that G4 and iM structures may have opposing functions in the control of oncogene transcription: while G4 formation and its ligand-induced stabilization generally inhibits gene expression, stabilization of iM seems to have transcription activating capabilities (Kang et al., 2014; Kendrick et al., 2014). Therefore, a molecule that is able to stabilize a G4 structure and to destabilize an iM structure may exert a synergistic effect on the inhibition of transcription. These are the cases of BRACO-19 (**2**), Mitoxantrone (**3**), and Phen-DC3 (**4**), whose biological activity may be ascribed to both mechanisms.

Overall, the present study highlights the necessity, when studying G4-targeting compounds, of evaluating also their effects on the i-motif counterparts, especially if one is looking for a “specific” drug.

## Author contributions

AR, JA, BP, and ZW conceived and designed the experiments. AP, NI, EG, and AD performed the CD, UV, and FRET experiments. DB performed the NMR experiments. MA performed the FID experiments. AP and JA carried out the synthesis of oligonucleotides. AP, NI, MA, EN, ZW, BP, JA, and AR analyzed the results. AR, JA, and ZW wrote the paper. All authors verified the data, contributed to the manuscript, and approved the final version.

### Conflict of interest statement

The authors declare that the research was conducted in the absence of any commercial or financial relationships that could be construed as a potential conflict of interest.
