# Supplementary material for: Common G-Quadruplex Binding Agents Found to Interact With i-Motif-Forming DNA: Unexpected Multi-Target-Directed Compounds
Source: Front Chem. 2018 Jul 24;6:281. doi: 10.3389/fchem.2018.00281 (PMC6066642; doi:10.3389/fchem.2018.00281)
Supplement: Supplementary file 1 [file Presentation_1.PDF]

# Common G-quadruplex binding agents found to interact with i-motif-forming DNA: unexpected multi-target-directed compounds

Alessia Pagano,[1,†] Nunzia Iaccarino,[1,†] Mahmoud A. S. Abdelhamid,[2] Diego Brancaccio,[1] Emanuele U. Garzarella,[1] Anna Di Porzio,[1] Ettore Novellino,[1] Zoë A. E. Waller,[2,3] Bruno Pagano,[1] Jussara Amato[1,*] and Antonio Randazzo[1,*]

[1] *Department of Pharmacy, University of Naples Federico II, Naples, Italy*
[2] *School of Pharmacy, University of East Anglia, Norwich Research Park, Norwich, UK*
[3] *Centre for Molecular and Structural Biochemistry, University of East Anglia, Norwich Research Park, Norwich, UK*

# Supplementary Content

[†] These authors contributed equally to this work.

*Corresponding Authors
*E-mail* addresses: jussara.amato@unina.it (J.A.), antonio.randazzo@unina.it (A.R.).

# Table of Contents


| Content | Page |
|---|---|
| **Figure S1.** $^{1}$H-NMR spectra of hTeloC (0.2 mM) in 10 mM $NaH_2PO_4$ at different pH values. | 2 |
| **Figure S2.** CD spectra of hTeloC (10 µM) in 10 mM $NaH_2PO_4$ at different pH values. | 3 |
| **Figure S3.** CD spectra of mutTel24 (10 µM) in 10 mM $KH_2PO_4$ at pH 4.3 with different ligands. | 4 |
| **Figure S4.** CD spectra of mutTel24 (10 µM) in 10 mM $KH_2PO_4$ at pH 5.7 with different ligands. | 4 |
| **Figure S5.** CD spectra of hTeloC (10 µM) in 10 mM $NaH_2PO_4$ at pH 4.3 with different ligands. | 5 |
| **Figure S6.** CD spectra of hTeloC (15 µM) in 10 mM $NaH_2PO_4$ at pH 5.7 with different ligands. | 5 |
| **Figure S7.** CD melting of mutTel24 (10 µM) in 10 mM $KH_2PO_4$ at pH 4.3 with different ligands at 290 nm. | 6 |
| **Figure S8.** CD melting of mutTel24 (10 µM) in 10 mM $KH_2PO_4$ at pH 5.7 with different ligands at 290 nm. | 6 |
| **Figure S9.** CD melting of hTeloC (10 µM) in 10 mM $NaH_2PO_4$ at pH 4.3 with different ligands at 288 nm. | 7 |
| **Figure S10.** CD melting of hTeloC (15 µM) in 10 mM $NaH_2PO_4$ at pH 5.7 with different ligands at 288 nm. | 7 |
| **Figure S11.** UV melting of mutTel24 (10 µM) in 10 mM $KH_2PO_4$ at pH 4.3 with different ligands at 295 nm. | 8 |
| **Figure S12.** UV melting of mutTel24 (10 µM) in 10 mM $KH_2PO_4$ at pH 5.7 with different ligands at 295 nm. | 8 |
| **Figure S13.** UV melting of hTeloC (10 µM) in 10 mM $NaH_2PO_4$ at pH 4.3 with different ligands at 295 nm. | 9 |
| **Figure S14.** UV melting of hTeloC (10 µM) in 10 mM $NaH_2PO_4$ at pH 5.7 with different ligands at 295 nm. | 9 |
| **Figure S15.** FRET melting of G4-F21T (0.1 µM) in 10 mM $KH_2PO_4$ at pH 5.7 with different ligands. | 10 |
| **Figure S16.** FRET melting of iM-F24T (0.1 µM) in 10 mM $NaH_2PO_4$ at pH 5.7 with different ligands. | 10 |
| **Figure S17.** Dose response curves from FID assays of $mutTel24_{FID}$ (1 µM) in 10 mM $KH_2PO_4$ at pH 4.3 and $hTeloC_{FID}$ (1 µM) in 10 mM $NaH_2PO_4$ at pH 4.3 with different ligands. | 11 |
| **Figure S18.** Dose response curves from FID assays of $mutTel24_{FID}$ (1 µM) in 10 mM $KH_2PO_4$ at pH 5.7 and $hTeloC_{FID}$ (1 µM) in 10 mM $NaH_2PO_4$ at pH 5.7 with different ligands. | 11 |
| **Figure S19.** Computed microspecies distribution (%) for BRACO-19 (A) and Pyridostatin (B) in the pH range 3.5-7.5. The structures of the molecules in the different protonation states are also shown. | 12 |

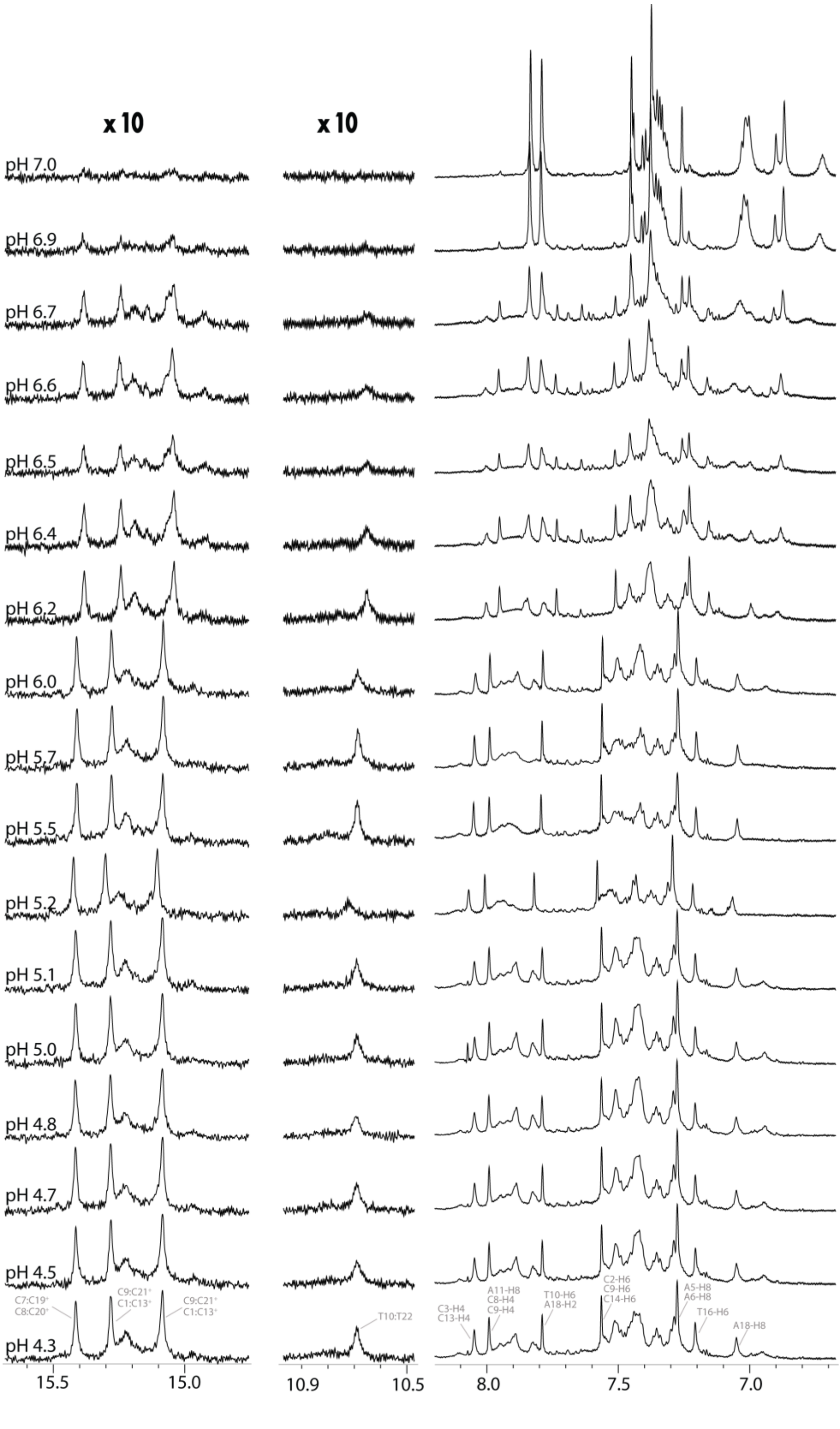


**Figure S1** $^{1}$H-NMR spectra of hTeloC (0.2 mM) in 10 mM $NaH_2PO_4$ at different pH values.

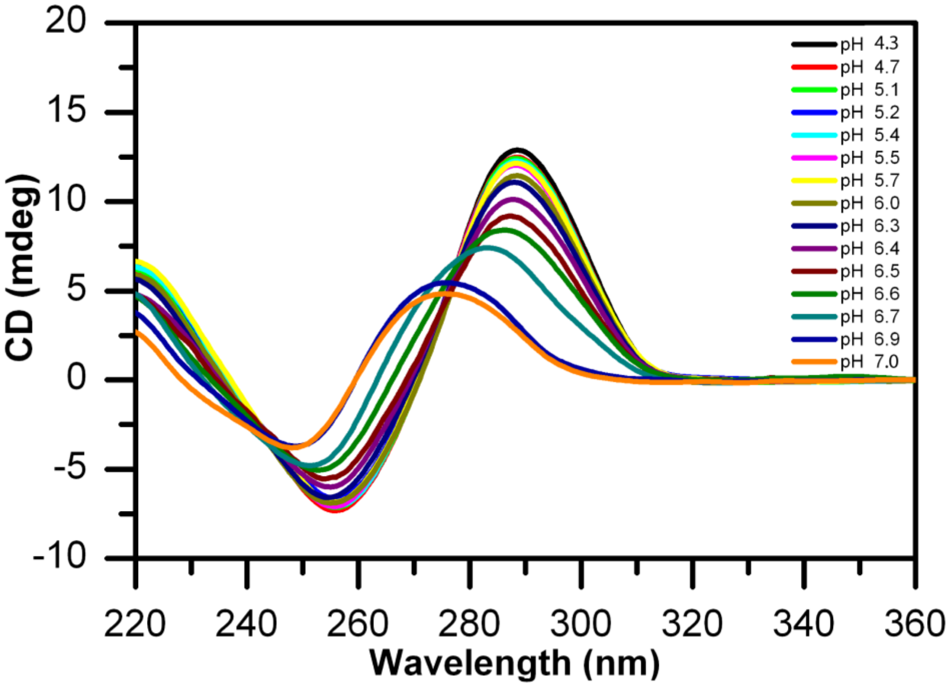


**Figure S2** CD spectra of hTeloC (10 µM) in 10 mM $NaH_2PO_4$ at different pH values.

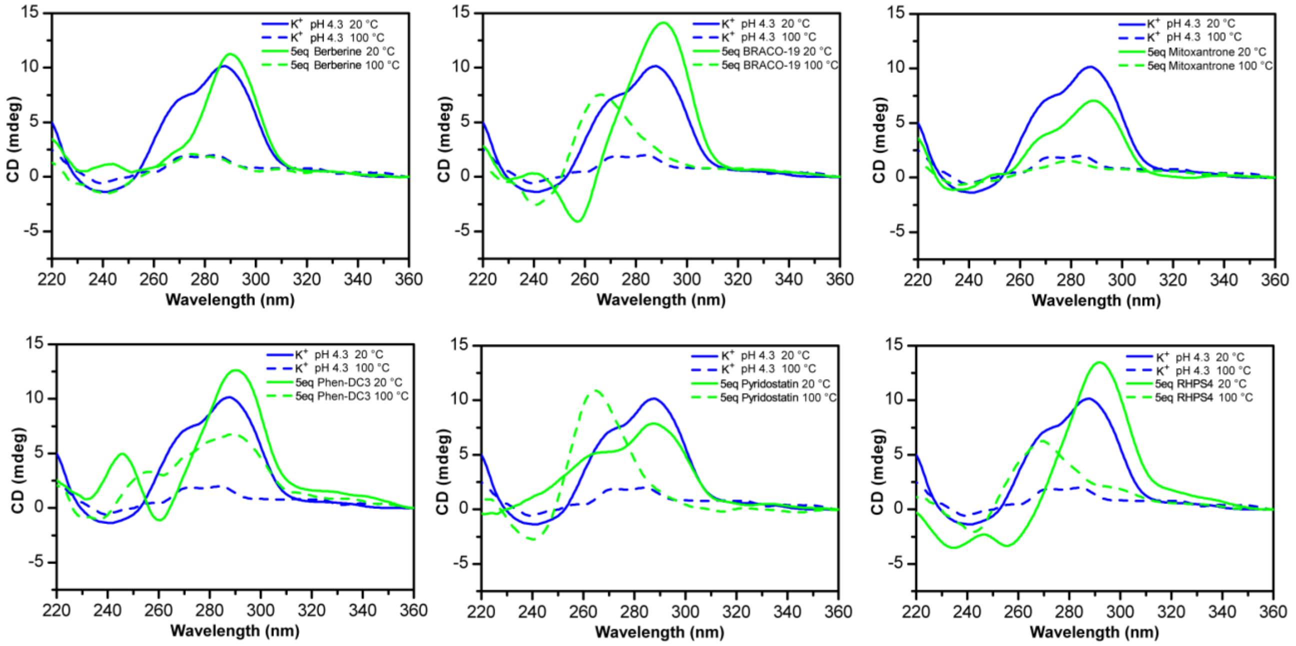


**Figure S3** CD spectra of mutTel24 (10 μM) in 10 mM $KH_2PO_4$ at pH 4.3 with different ligands.

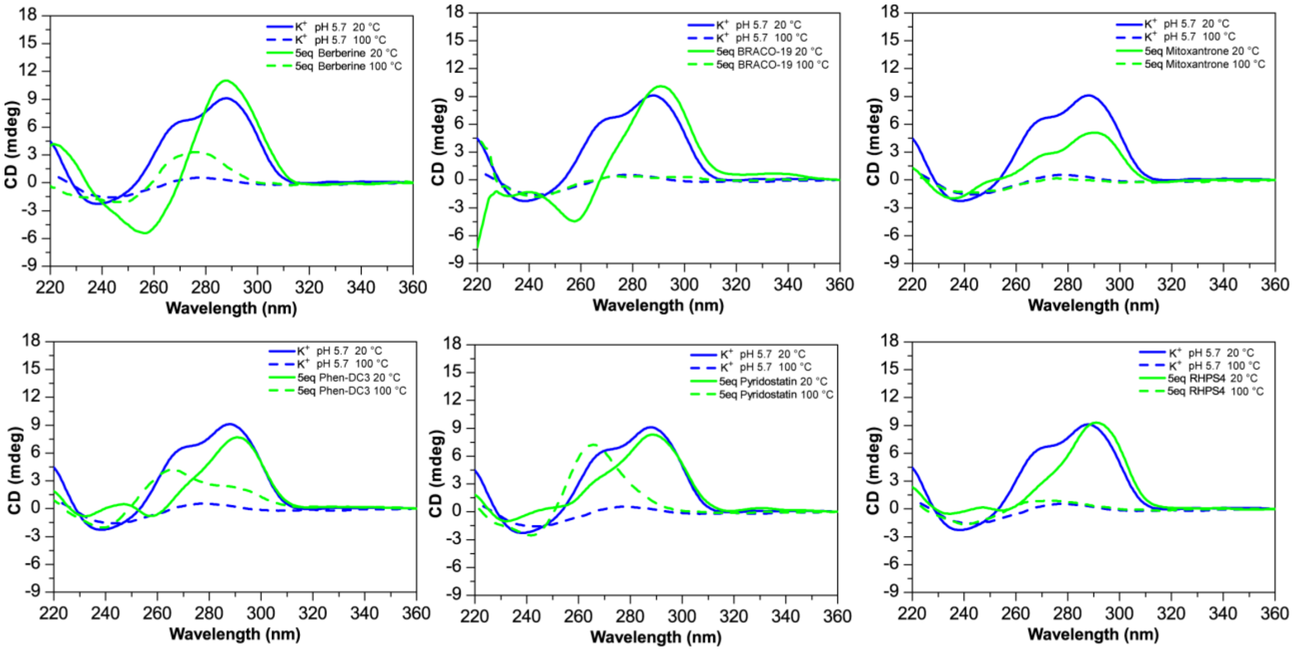


**Figure S4** CD spectra of mutTel24 (10 μM) in 10 mM $KH_2PO_4$ at pH 5.7 with different ligands.

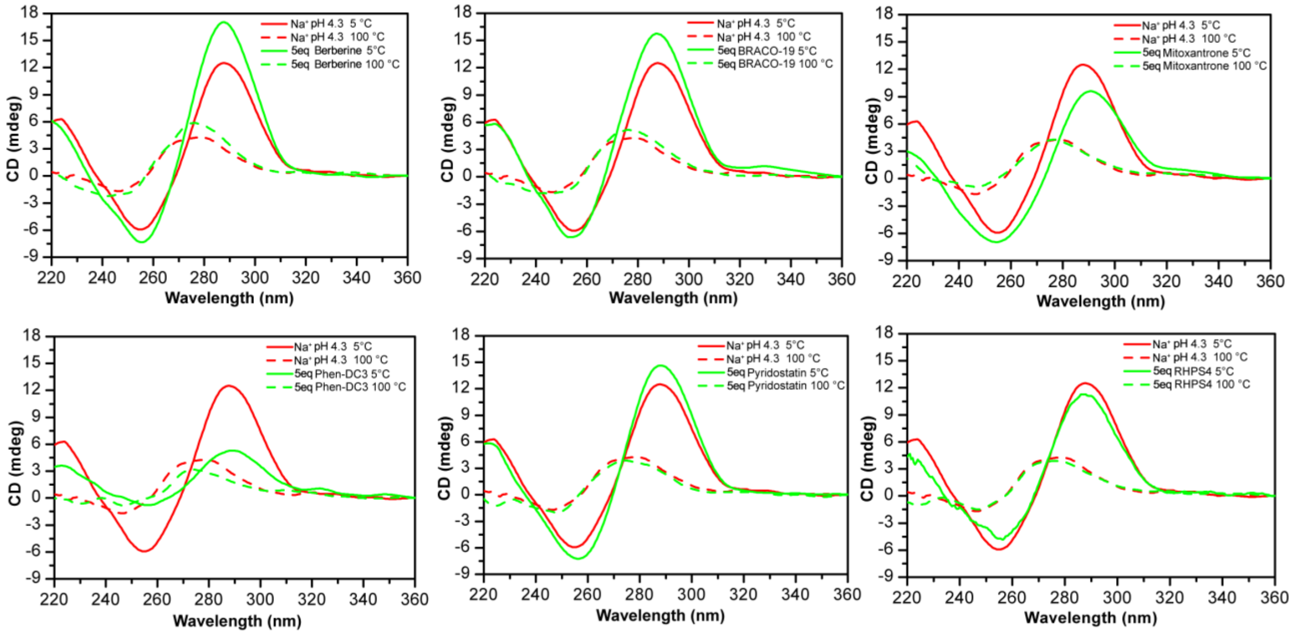


**Figure S5** CD spectra of hTeloC (10 µM) in 10 mM $NaH_2PO_4$ at pH 4.3 with different ligands.

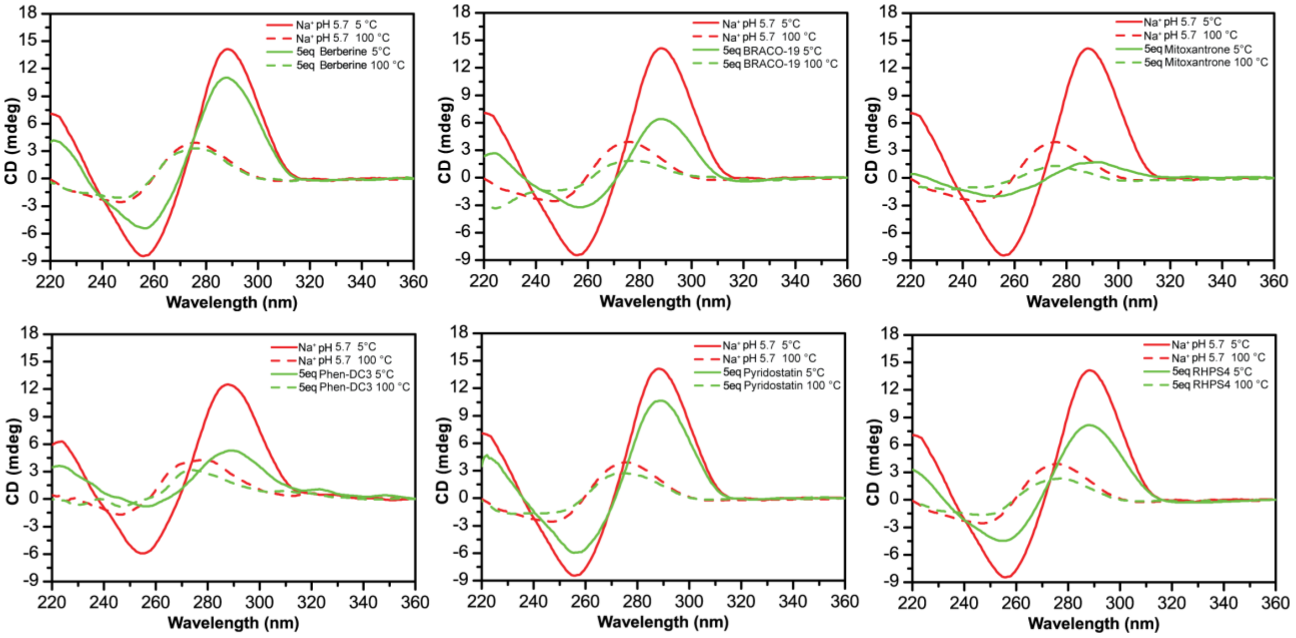


**Figure S6** CD spectra of hTeloC (15 µM) in 10 mM $NaH_2PO_4$ at pH 5.7 with different ligands.

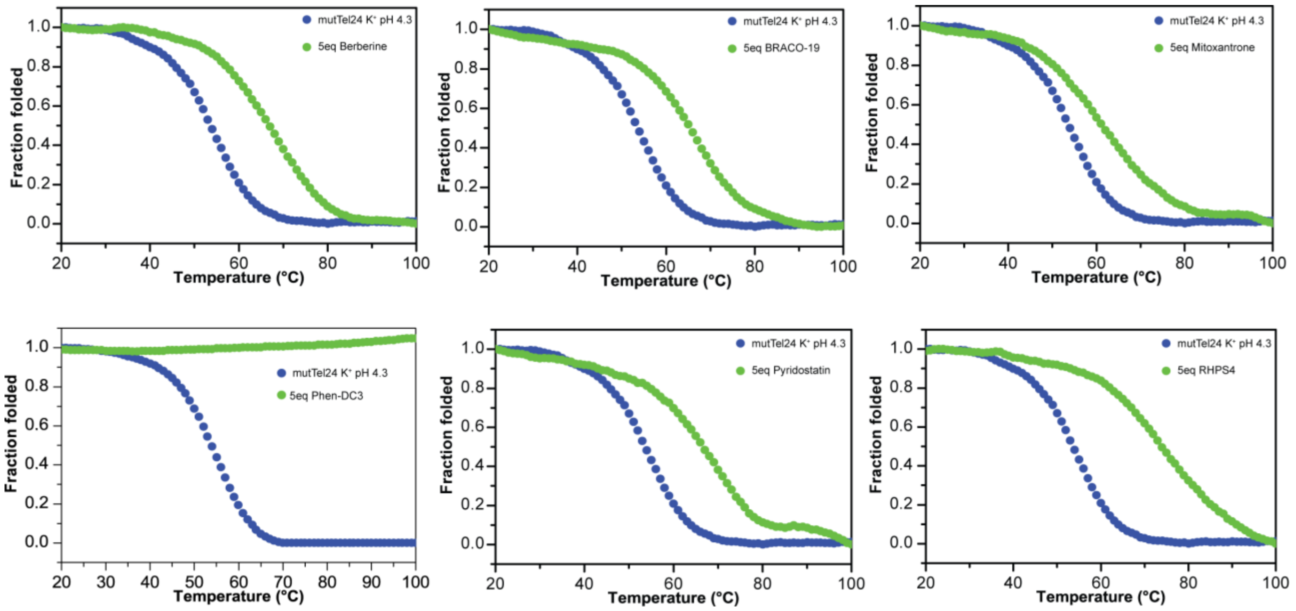


**Figure S7** CD melting of mutTel24 (10 µM) in 10 mM $KH_2PO_4$ at pH 4.3 with different ligands at 290 nm.

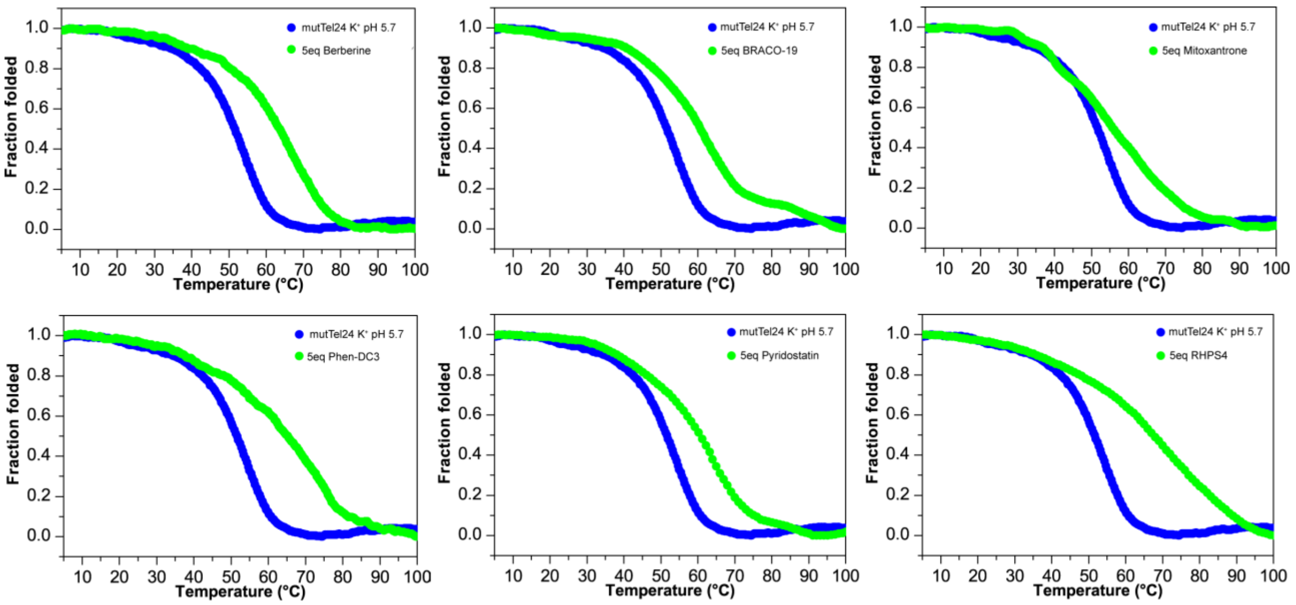


**Figure S8** CD melting of mutTel24 (10 µM) in 10 mM $KH_2PO_4$ at pH 5.7 with different ligands at 290 nm.

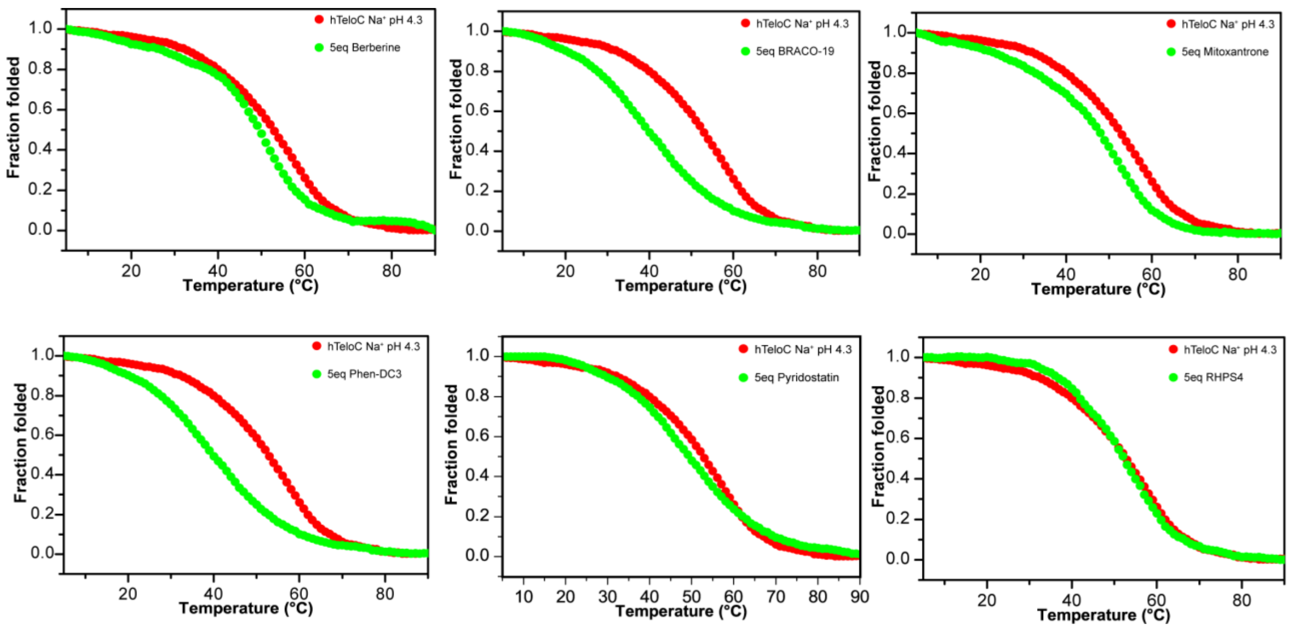


**Figure S9** CD melting of hTeloC (10 µM) in 10 mM $NaH_2PO_4$ at pH 4.3 with different ligands at 288 nm.

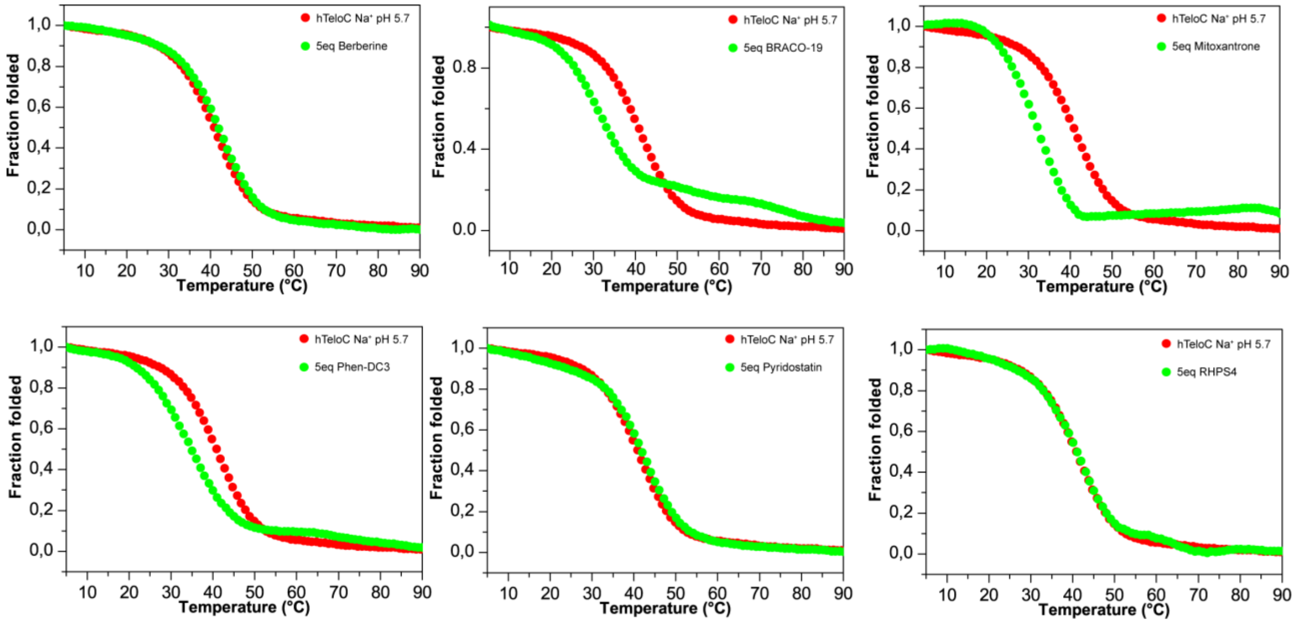


**Figure S10** CD melting of hTeloC (15 µM) in 10 mM $NaH_2PO_4$ at pH 5.7 with different ligands at 288 nm.

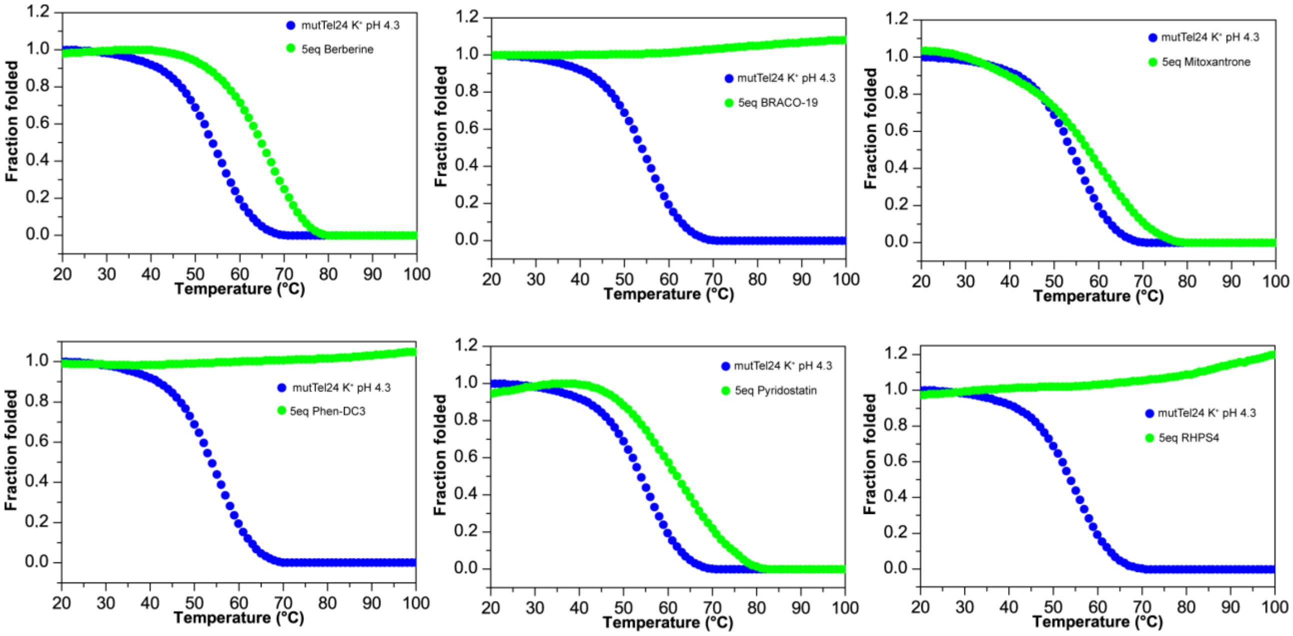


**Figure S11** UV melting of mutTel24 (10 µM) in 10 mM $KH_2PO_4$ at pH 4.3 with different ligands at 295 nm.

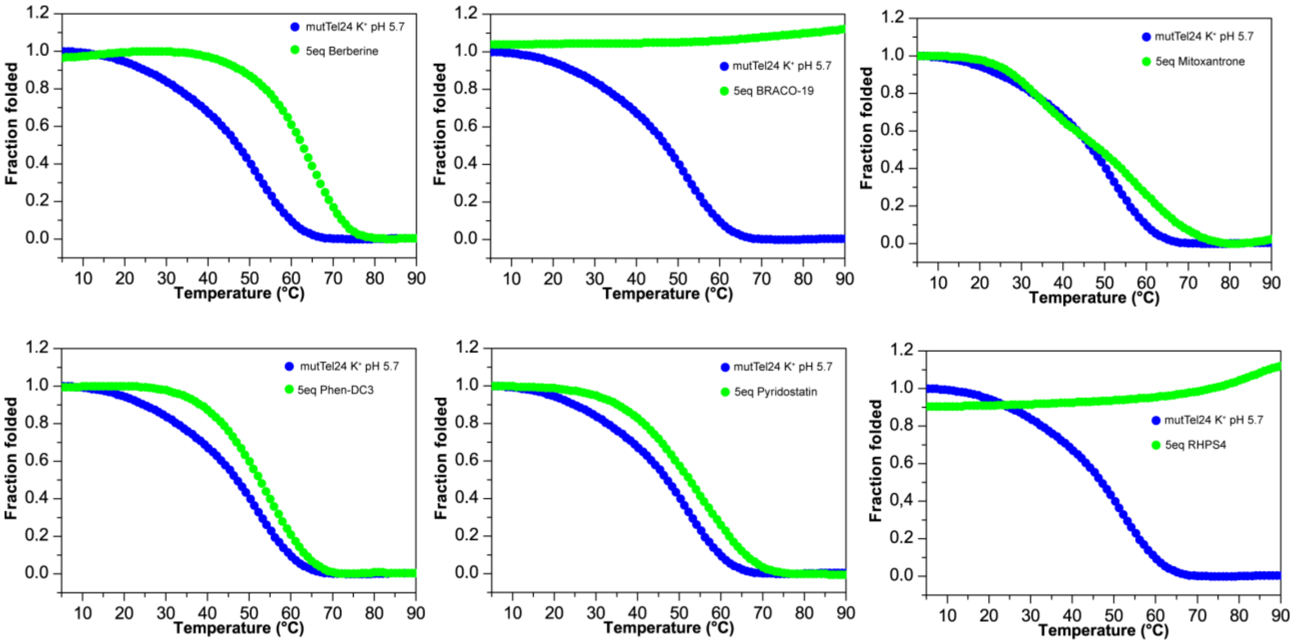


**Figure S12** UV melting of mutTel24 (10 µM) in 10 mM $KH_2PO_4$ at pH 5.7 with different ligands at 295 nm.

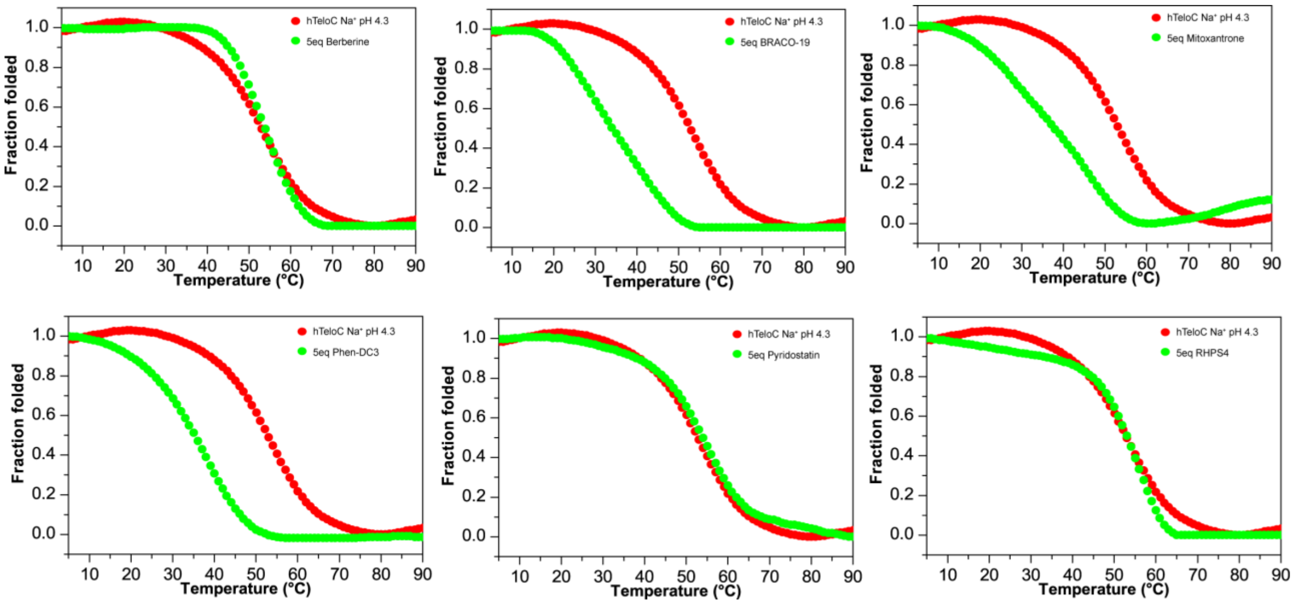


**Figure S13** UV melting of hTeloC (10 µM) in 10 mM $NaH_2PO_4$ at pH 4.3 with different ligands at 295 nm.

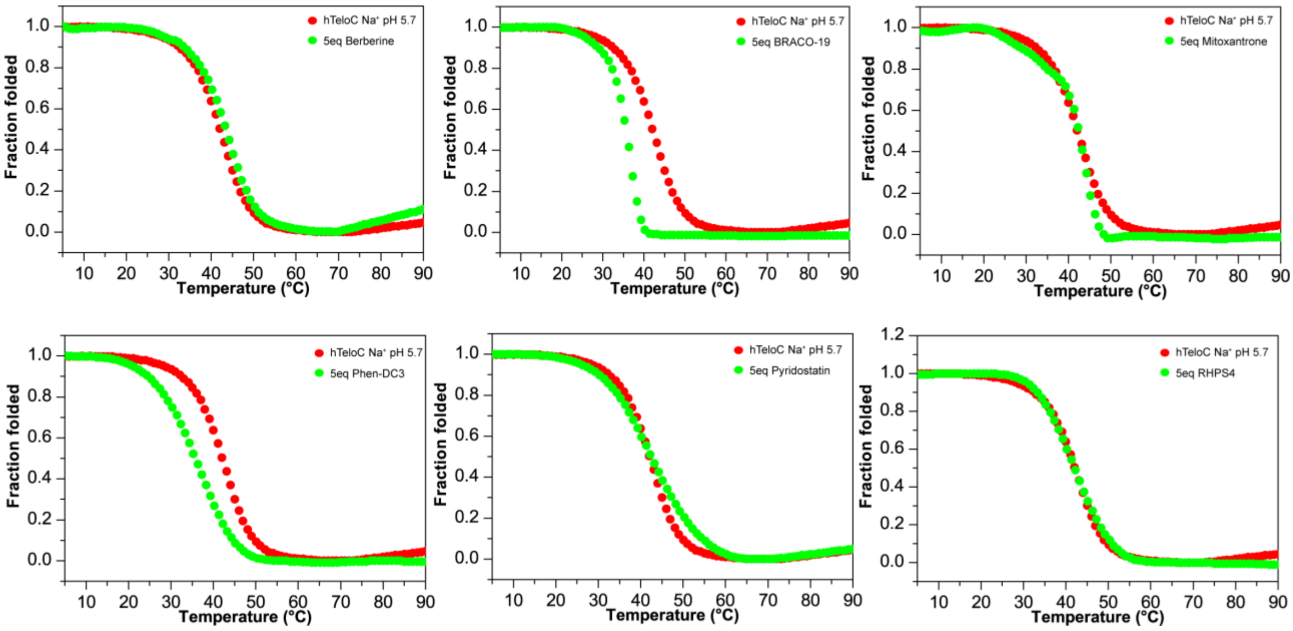


**Figure S14** UV melting of hTeloC (10 µM) in 10 mM $NaH_2PO_4$ at pH 5.7 with different ligands at 295 nm.

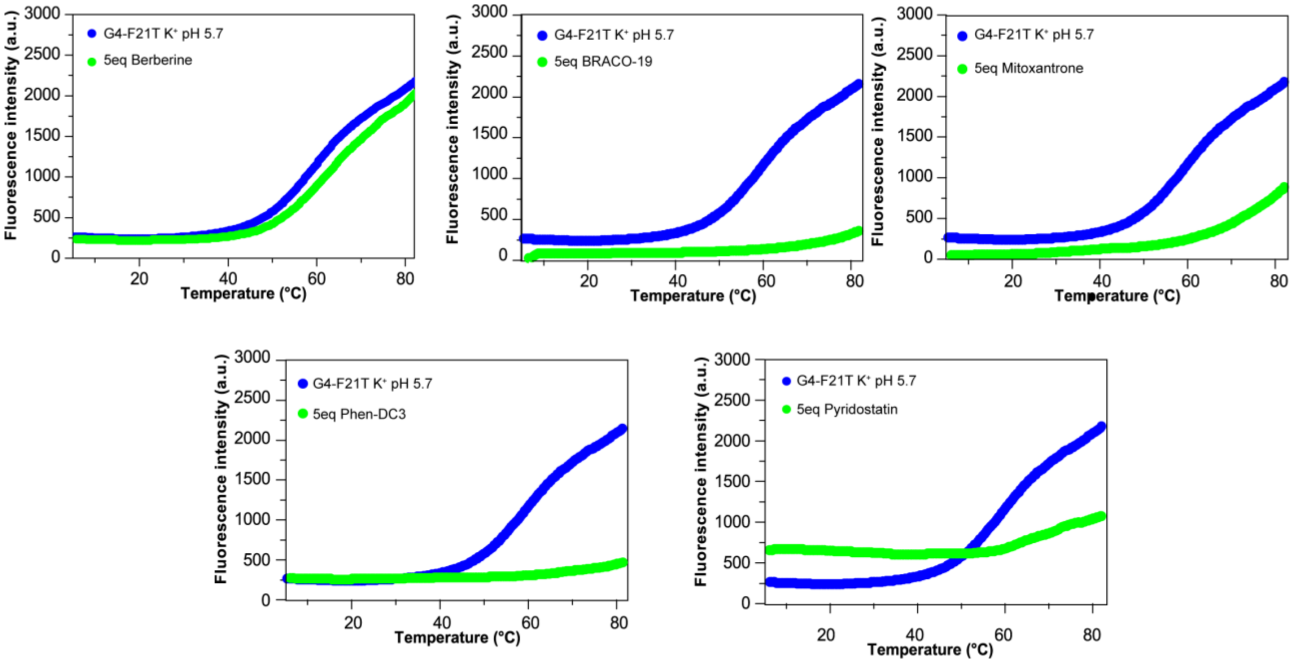


**Figure S15** FRET melting of G4-F21T (0.1 μM) in 10 mM $KH_2PO_4$ at pH 5.7 with different ligands.

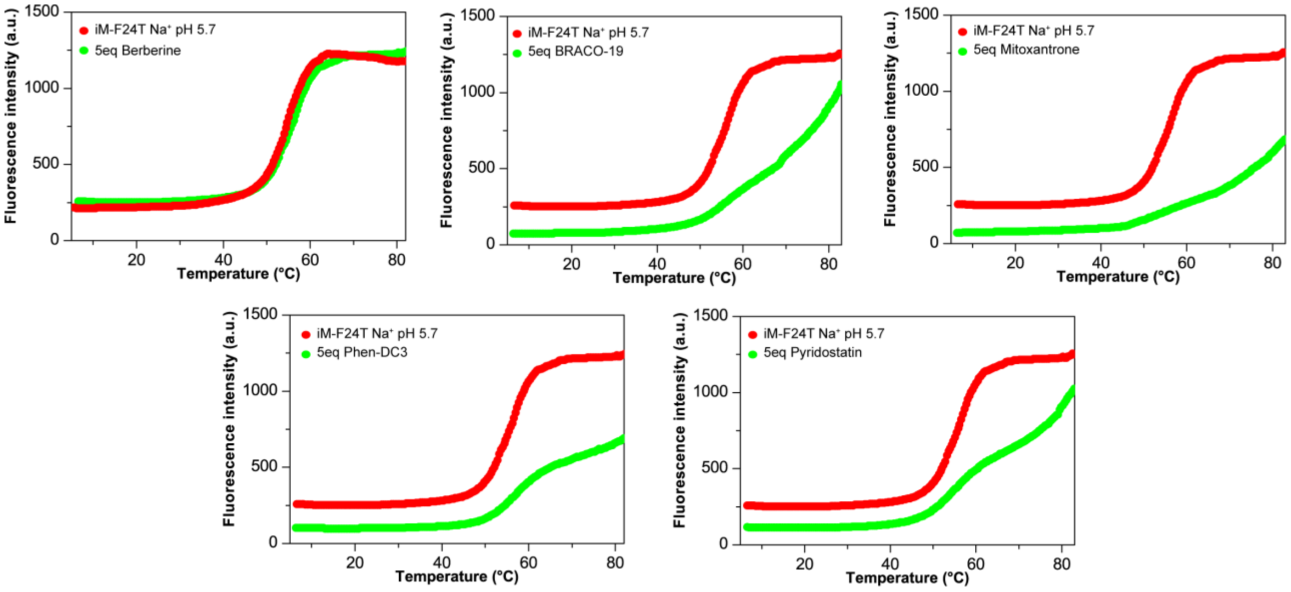


**Figure S16** FRET melting of iM-F24T (0.1 μM) in 10 mM $NaH_2PO_4$ at pH 5.7 with different ligands.

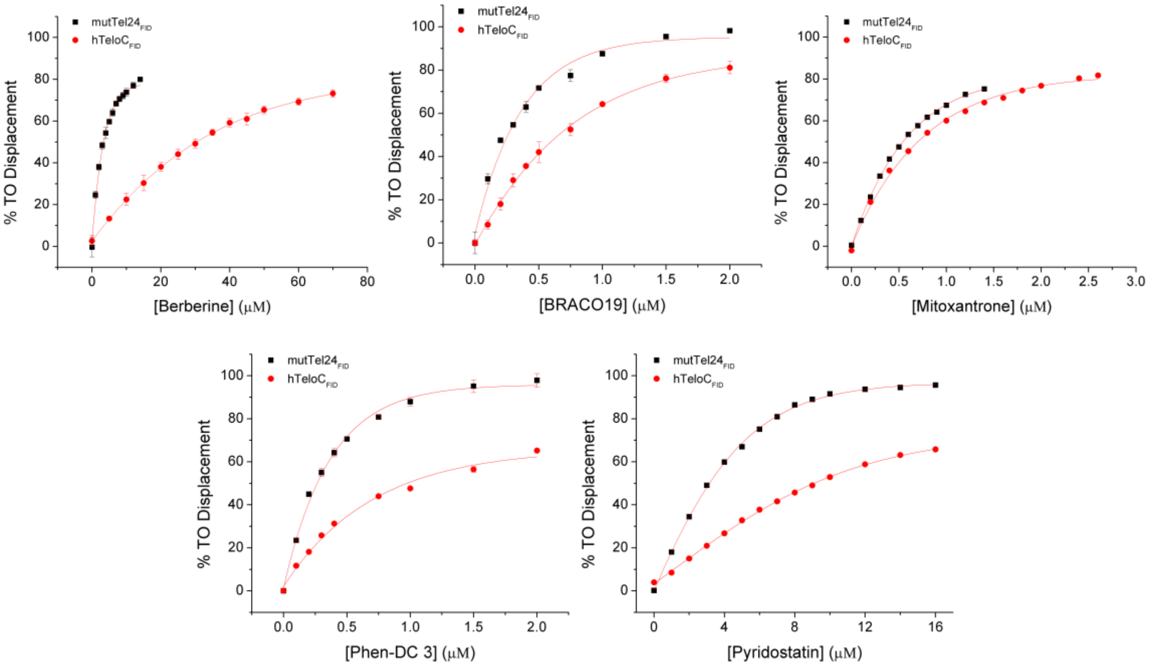


**Figure S17** Dose response curves from FID assays of mutTel24$_{FID}$ (1 µM) in 10 mM $KH_2PO_4$ at pH 4.3 and hTeloC$_{FID}$ (1 µM) in 10 mM $NaH_2PO_4$ at pH 4.3 with different ligands.

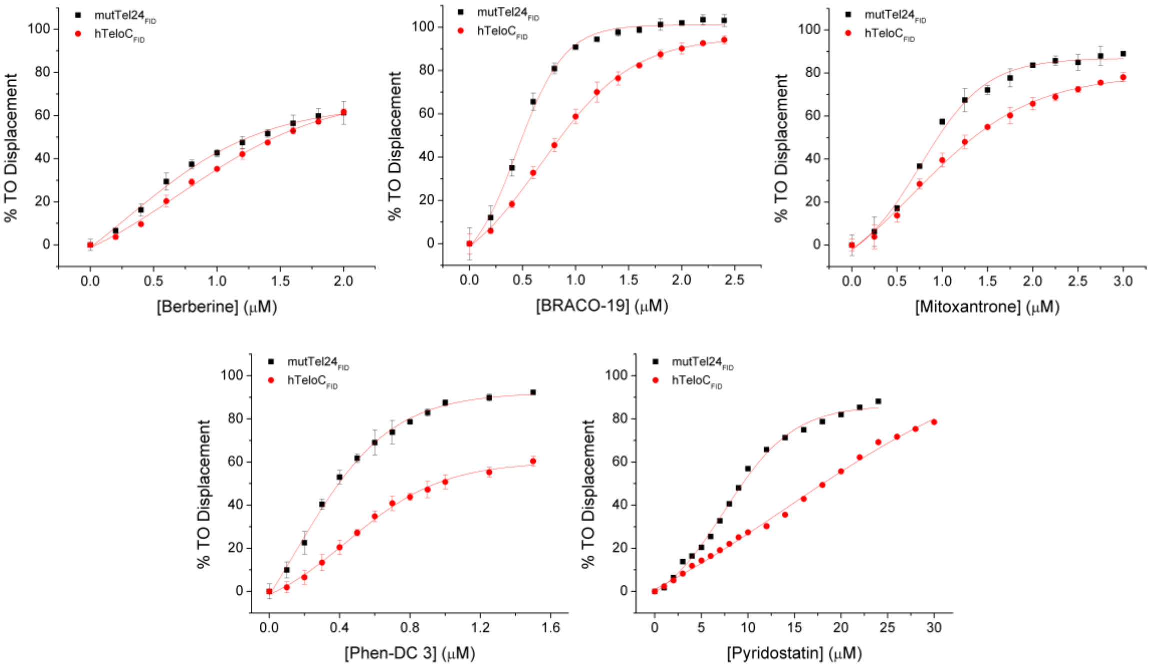


**Figure S18** Dose response curves from FID assays of mutTel24$_{FID}$ (1 µM) in 10 mM $KH_2PO_4$ at pH 5.7 and hTeloC$_{FID}$ (1 µM) in 10 mM $NaH_2PO_4$ at pH 5.7 with different ligands.

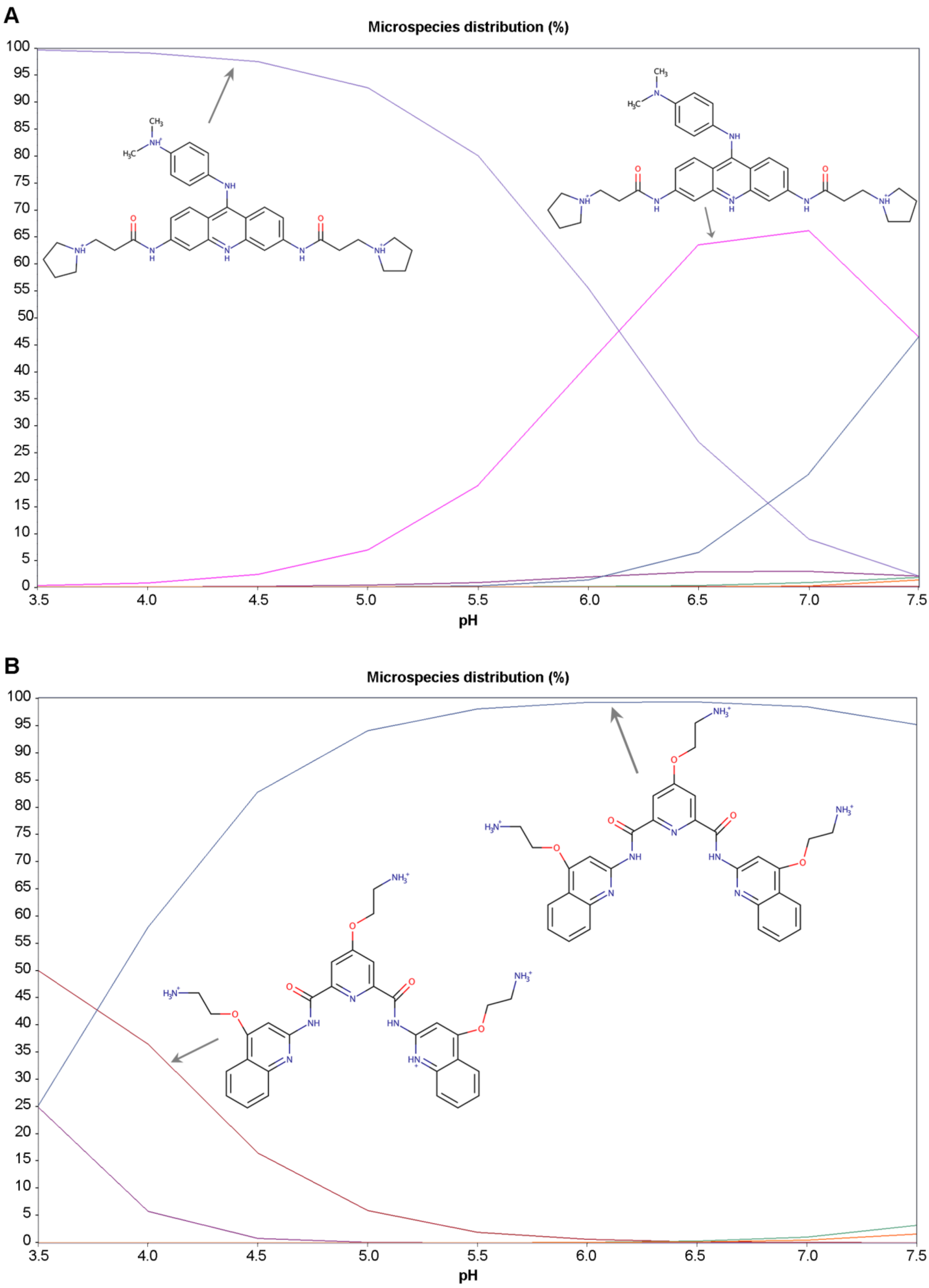


**Figure S19** Computed microspecies distribution (%) for BRACO-19 (A) and Pyridostatin (B) in the pH range 3.5-7.5. The structures of the molecules in the different protonation states are also shown.
